# A new class of peptides from wasp venom: a pathway to antiepileptic/neuroprotective drugs

**DOI:** 10.1093/braincomms/fcad016

**Published:** 2023-02-17

**Authors:** Márcia Renata Mortari, Alexandra O S Cunha, Lilian C dos Anjos, Henrique O Amaral, Maria Varela Torres Quintanilha, Erica A Gelfuso, Mauricio Homem-de-Mello, Hugo de Almeida, Solange Rego, Bernard Maigret, Norberto P Lopes, Wagner F dos Santos

**Affiliations:** Neuropharmacology Laboratory, Department of Physiological Sciences, Institute of Biological Sciences, University of Brasília, Brasília 71910-900, Brazil; Neurobiology and Venoms Laboratory, Department of Biology, Faculty of Philosophy, Sciences and Literature of Ribeirão Preto, University of São Paulo, São Paulo 14040-900, Brazil; Neurobiology and Venoms Laboratory, Department of Biology, Faculty of Philosophy, Sciences and Literature of Ribeirão Preto, University of São Paulo, São Paulo 14040-900, Brazil; Neuropharmacology Laboratory, Department of Physiological Sciences, Institute of Biological Sciences, University of Brasília, Brasília 71910-900, Brazil; Neuropharmacology Laboratory, Department of Physiological Sciences, Institute of Biological Sciences, University of Brasília, Brasília 71910-900, Brazil; Neuropharmacology Laboratory, Department of Physiological Sciences, Institute of Biological Sciences, University of Brasília, Brasília 71910-900, Brazil; Neuropharmacology Laboratory, Department of Physiological Sciences, Institute of Biological Sciences, University of Brasília, Brasília 71910-900, Brazil; in Silico Toxicology Laboratory (inSiliTox), Department of Pharmacy, Health Sciences School, University of Brasilia, Brasilia 71910-900, Brazil; Team CAPSID, Laboratoire Lorrain de Recherche en Informatique et ses applications (LORIA), Vandoeuvre Les Nancy F-54506, France; Team CAPSID, Laboratoire Lorrain de Recherche en Informatique et ses applications (LORIA), Vandoeuvre Les Nancy F-54506, France; Team CAPSID, Laboratoire Lorrain de Recherche en Informatique et ses applications (LORIA), Vandoeuvre Les Nancy F-54506, France; Organic Chemistry Laboratory, Department of Physics and Chemistry, Faculty of Pharmaceutical Sciences of Ribeirão Preto, University of São Paulo, São Paulo 14040-900, Brazil; Neurobiology and Venoms Laboratory, Department of Biology, Faculty of Philosophy, Sciences and Literature of Ribeirão Preto, University of São Paulo, São Paulo 14040-900, Brazil

**Keywords:** Wasp venoms, neuropeptides, antiepileptic drug, neuroprotection, epilepsy

## Abstract

The ability of venom-derived peptides to disrupt physiological processes in mammals provides an exciting source for pharmacological development. Our research group has identified a new class of neuroactive peptides from the venom of a Brazilian social wasp, *Polybia occidentalis*, with the potential pharmacological profile to treat epilepsies. The study was divided into five phases: Phase 1 concerned the extraction, isolation and purification of Occidentalin-1202(n) from the crude venom, followed by the synthesis of an identical analogue peptide, named Occidentalin-1202(s). In Phase 2, we described the effects of both peptides in two acute models of epilepsy—kainic acid and pentylenetetrazole-induced model of seizures—and measured estimated ED_50_ and therapeutic index values, electroencephalographic studies and C-fos evaluation. Phase 3 was a compilation of advanced tests performed with Occidentalin-1202(s) only, reporting histopathological features and its performance in the pilocarpine-induced *status epilepticus*. After the determination of the antiepileptic activity of Occidentalin-1202(s), Phase 4 consisted of evaluating its potential adverse effects, after chronic administration, on motor coordination (Rotarod) and cognitive impairment (Morris water maze) tests. Finally, in Phase 5, we proposed a mechanism of action using computational models with kainate receptors. The new peptide was able to cross the blood–brain barrier and showed potent antiseizure effects in acute (kainic acid and pentylenetetrazole) and chronic (temporal lobe epilepsy model induced by pilocarpine) models. Motor and cognitive behaviour were not adversely affected, and a potential neuroprotective effect was observed. Occidentalin-1202 can be a potent blocker of the kainate receptor, as assessed by computational analysis, preventing glutamate and kainic acid from binding to the receptor’s active site. Occidentalin-1202 is a peptide with promising applicability to treat epilepsy and can be considered an interesting drug model for the development of new medicines.

## Teaser

Mortari *et al*. report a new wasp venom-derived peptide capable of protecting against seizures induced in both acute and chronic models. This peptide provides a unique platform for the development of innovative treatments for epilepsy.

## Graphical Abstract

**Graphical abstract:**
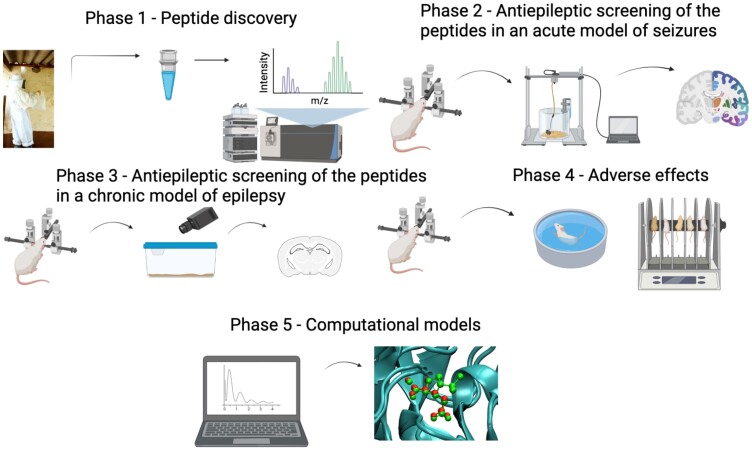

## Introduction

The use of components isolated from venoms for biomedical research has proven to be a great success, and their application has been directed toward different strategies, including the development of new research tools, diagnostic reagents and therapeutic drugs.^1–3^ Indeed, naturally occurring peptides (isolated from venoms) are an outstanding platform for drug development, mainly due to intrinsic and extrinsic factors that contribute to the effectiveness of these compounds, such as the ability to disrupt physiological processes in mammals, high specificity and affinity for receptors, and wide potency profile.^1,4,5^

The action mechanisms of the highly promising members of this neuroactive peptide group involve interactions with targets in the Central Nervous System (CNS) of mammals. They can operate in different parts of this system such as ionic channels, release and reuptake of neurotransmitters or even as an agonist or antagonist of receptors.^6–8^ In this context, neuroactive peptides isolated from arthropod venoms may be particularly useful to antiepileptic drug (AED) research, since epilepsy involves neuronal substrates, which are the sites of action of many neurotoxins.^9,10^ Social wasp venoms are composed of several peptides with diverse pharmacological actions. Interestingly, we evaluated the denatured crude venom of *Polybia occidentalis* and this crude venom revealed a potent anticonvulsant effect against seizures induced by four distinct convulsants: bicuculline, picrotoxin, kainic acid (KA) and pentylenetetrazole (PTZ).^11^ Moreover, the denatured venom of other social wasps provokes a decrease in the uncompetitive uptake of GABA and glutamate in rat cerebral cortex synaptosomes.^12,13^ However, the chemical characterization of the active compound has not been established yet.

Epilepsy is a common and devastating disease of the brain, characterized by spontaneous and repeated unprovoked epileptic seizures,^14,15^ affecting 1% of the world’s population.^16^ Treatment for this disease is based on the chronic administration of AEDs,^17,18^ but they often have intolerable side-effects,^19–21^ which vary in frequency and severity and may include chronic toxicity, sedation, cognitive impairment, aplastic anaemia, teratogenicity and hepatic failure.^22^ Besides, failure to treat epilepsy can cause neurobiological, cognitive and psychosocial consequences and a reduction in the patient’s quality of life.^23–26^

In the present work, we isolated and characterized a novel class of antiepileptic peptides from the venom of a social wasp, *P. occidentalis*. We also assessed the neuropharmacological profile of the natural peptide, named Occidentalin-1202(n), and synthetic peptide, named Occidentalin-1202(s), using the acute and chronic model of chemoconvulsant-induced seizures, and evaluated possible adverse effects. Furthermore, we performed immunohistochemical and docking analysis to investigate the possible mechanism of action for Occidentalin-1202(s).

## Materials and methods

### Phase 1: Peptide discovery

#### Venom extracts

The venom extracts were collected from females of *P. occidentalis* at the University of São Paulo (Campus of Ribeirão Preto City, São Paulo, Brazil). The wasps were sacrificed by freezing at −20°C. After species identification, the venom reservoirs were manually removed, crushed in Milli-Q grade water/50% acetonitrile and centrifuged at 5000*×g* for 10 min at 4°C. Supernatants were carefully collected and filtered using Microcon 3 (Millipore). The extract with compounds lower than 3 kDa was obtained, lyophilized and weighed.

#### Fractionation and purification

The extracts were resuspended in 2% acetonitrile/water (ACN/H_2_O) and 0.07% trifluoroacetic acid (TFA). The solution was chromatographed on a reverse-phase high-performance liquid chromatography (RP-HPLC) column (C18 ODS, Jupiter 15 µm, 4.6 × 250 mm, Phenomenex, Torrence, CA, USA) at a flow rate of 5.0 ml/min. The elution started with a gradient of 2% ACN (containing 0.07% TFA) for 20 min, followed by a variation from 2 to 60% ACN (containing 0.07% TFA) in 40 min, and then 60% ACN (containing 0.07% TFA) in 20 min. The active fraction was re-chromatographed on an RP-HPLC column (C18 ODS, Jupiter 5 µm, 4.6 × 150 mm, Phenomenex, Torrence, CA, USA) at a flow rate of 1.0 ml/min. Elution was performed using a 2:98: ACN:H_2_O (containing 0.07% TFA) mobile phase for 10 min, followed by a gradient varying from 2 to 60% ACN (containing 0.07% TFA) for 30 min, and 60% ACN (containing 0.07% TFA) for 10 min. Both runs were monitored at 214 nm.

#### Electrospray mass spectrometry

Molecular mass spectral analyses of peptides were performed on high-resolution quadrupole-time-of-flight mass spectrometry (q-TOF). Electrospray Ionisation Mass Spectrometry (ESI-MS) spectrum was acquired on a quadrupole time-of-ﬂight instrument (UltrOTOF apparatus, Bruker Daltonics, Billerica, USA).

#### Peptide sequencing by MS/MS

A Quattro-LC instrument from Micromass (Manchester, UK) was used for peptide sequencing. Solutions were infused into the ESI source at 10 µl/min using a Harvard Apparatus syringe pump model 1746 (Holliston, MA, USA). Ultrapure water (Milli-Q) was used throughout the study. The mass scan range was from *m*/*z* 1000–2000. Experiments were performed with a cone voltage of 30 V, a capillary voltage of 3 kV and a desolvation gas temperature of 80°C. A single-charged (protonated) molecule was submitted to collision-induced dissociation (CID) with argon gas at 10–50 eV collision energies. All MS/MS experiments were performed using continuous acquisition mode, scanning from *m*/*z* 50–2000, with a scan time of 5 s.

Peptide sequencing was obtained using the ion products described in the mass spectra of each peptide, with the aid of AminoCalc software (Protana A/S). Primary sequences obtained were compared with other sequences available in databases by a program in Expasy server (Expasy Molecular Biology server; http://www.expasy.org) and BLASTP (https://www.uniprot.org/blast/).

#### Peptide synthesis and purification

An analogue of the natural peptide was synthesized manually by the solid-phase peptide method on a 4-methyl-benzhydryl amine-resin (0.8 mmol/g), using the t-Boc strategy. Full deprotection and cleavage of the peptide from the resin were carried out using anhydrous HF treatment with anisole and dimethyl sulphide as scavengers at 0°C for 1.5 h. The resulting peptide solution was kept at pH 6.8–7.0 and 5°C for 72 h. The purification and sequence identification of Occidentalin-1202(s) followed the same methods as previously described.

#### Animals and ethical principles

Male Wistar rats (*Rattus norvegicus*; 220–250 g) and Swiss mice (*Mus musculus*; 16–20 g) were used in the assays (*n* = 113, and *n* = 86, respectively). Only male animals were used to avoid any effects that sex and the oestrous cycle might have on the probing responses. The animals were kept in pairs in wire-mesh cages in a room with a 12 h dark/light cycle (lights on at 7:00 am) with food and water *ad libitum*. Rats were used in intracerebroventricular (i.c.v.) administration experiments, whereas mice were chosen for intraperitoneal (i.p.) administration of the peptide. Animal handling followed the ethical principles from the National Council for the Control of Animal Experimentation-CONCEA and the Arouca Law (Law n° 11.794/2008). The Committee for Ethics in Animal-CEUA of the University of Brasília (protocols *n*° 45.810/2009) approved all experimental procedures. Also, every effort was made to avoid unnecessary stress and pain to the experimental animals. The Brazilian Institute of Environment and Renewable Natural Resources allowed wasps collection (IBAMA Permit number: 21723-2). The National Council of Technological and Scientific Development provided the Access and Remittance Authorization for the Genetic Patrimony Component Use for Scientific Research (CNPq n° 010476/2013-0).

### Phase 2: Acute models of epilepsy

#### Stereotaxic surgery

Rats were anesthetized with ketamine (100 mg/kg) and xylazine 10 mg/kg (Cristália, Brazil) for stereotaxic implantation of a stainless-steel guide cannula (10 mm) in the cerebral right lateral ventricle. The coordinates used were anteroposterior (AP): 0.9 mm to bregma, mesolateral (ML): 1.6 mm and dorsal–ventral (DV): 3.4 mm ventral from the surface of the skull, according to the Paxinos and Watson’s atlas (1986). The cannula was fixed to the skull with dental acrylate and was sealed with stainless-steel wire to avoid obstruction.

The animals were allowed to rest for 4–6 days to recover from surgery. After that, the rats were gently wrapped in a cloth, hand-held and a thin dental needle (tribiselated and siliconized, 30 G, 10.2 mm length) was introduced through the guide cannula. The injection needle was linked to a polyethylene tube connected to a 10 µl Hamilton syringe, held by an infusion pump (Insight, Brazil) that injected a volume of 3 µl during 60 s.

#### Antiepileptic screening in acute models of epilepsy

All convulsants’ doses were based on previous experiments that searched for the dosage producing convulsions in 99% of the animals (CD_99_) (data not shown). We assessed the antiepileptic effect of the peptides in two different drug-induced acute models of epilepsy: the KA and PTZ models.

Before injections, animals were placed in the open field for 10 min, and after convulsant administration, they were recorded for 20 min. At the end of the filming, animals were packed in individual cages until total recovery.

Seizures induced by KA were scored according to the Pinel and Rovner (1978) scale.^27^ The latencies to the onset of seizures and percentages of the protected animals against score 8 seizures were the parameters used to analyse the anticonvulsant effects (% protection = *N* protected animals/*N* total × 100). Further, seizures induced by PTZ administration were analysed according to the Lamberty and Klitgaard (2000) index,^28^ considering the behaviour of convulsing animals, as well as the latencies to the onset of tonic-clonic seizures.

#### Antiepileptic screening by i.c.v. injection

In this test, rats were divided into groups (*n* = 4–6/group) and each group was treated with Occidentalin-1202(n) or Occidentalin-1202(s) by i.c.v. injection (3.00, 1.50, 0.30 and 0.15 μg/animal) 10 min before the administration of the convulsing drugs (KA 3.75 µg/animal, i.c.v. route and PTZ 100 mg/kg, injected via s.c.). The injection volume for all compounds via i.c.v. was 3 μl in 1 min, whereas PTZ was injected subcutaneously in an amount of 0.2 ml in the loose fold of the neck. Negative control groups were made for both convulsants, where the animals received pretreatment with the vehicle solution used to solubilize the peptide (1 μl of saline solution, i.c.v.; 0.2 ml s.c.).

#### Electroencephalographic studies

Electroencephalographic (EEG) seizures were obtained using video-electroencephalography (vEEG) manufactured by Pinnacle Technology, Inc. (Lawrence, KS, USA), which consists of a head-mounted platform and wire leads, preamplifier, commutator, mounting plate and a proprietary software including a data acquisition interfaced with a computer.

For vEEG monitoring, we performed a stereotaxic surgery identical to that described previously, except that the procedure involved affixing an EEG head mount with screws installed in the skull, and the corresponding EEG leads were wrapped around each screw. Six cortical electrodes were implanted on the dura mater over the cortex: two in the frontal region (AP = −1), two in the parietal region (AP = +2.5 mm) and two in the occipital region (AP = +6 mm) all in relation to bregma, 4 mm lateral distances to the midline. A stainless-steel guide cannula (10 mm in length and 26 G) was implanted in the right lateral ventricle. At the end of the surgery, everything was fixed to the skull surface with acrylic resin.

After five days from the surgery, rats (*n* = 3) were attached to a multichannel amplifier (Pinnacle Technologies 8400–9000 video/EEG system with Sirenia Software) by a flexible recording cable and an electric swivel, fixed above the cages, permitting free movements for the animals. Animals were kept in this system 30 min prior and 120 min following injections of KA (350 mg/kg, i.p.); 10 min before KA injections, animals received Occidentalin-1202(s) ED_100_ (3 µg/animal) or ED_50_ (0.4 µg/animal) or saline [phosphate-buffered saline (PBS)] as a single i.c.v. injection (ED_100_ and ED_50_ were previously calculated in the antiepileptic screening in acute models of epilepsy, see Table 1). All EEG records were performed starting at 9.00 am in order to avoid circadian alterations within groups. The animals were attached to a multichannel amplifier (Pinnacle Technology’s 8400–9000 video/EEG system with Sirenia Software, KS, USA) by a flexible recording cable and an electric swivel, fixed above the cages, permitting free movements for the animals. All EEG signals were amplified and conditioned by analogue filters (filtering: below 1 Hz and above 30 Hz at 6 dB/octave) and subjected to an analogue-to-digital conversion with a sampling rate of 250 Hz.

**Table 1:** KA and PTZ-induced seizure inhibition ED_50_ values of Occidentalin-1202(n) isolated from *P. occidentalis* venom and synthetic Occidentalin-1202(s)

Convulsant (dose)	Peptide	Animal	Administration route	ED_50_ (95% CI)
i.c.v. KA (2.4 μg/animal)	Occidentalin-1202(n) Occidentalin-1202(s)	Wistar rats	i.c.v. i.c.v.	0.33 (0.24–0.47) μg/animal 0.35 (0.18–0.65) μg/animal
s.c. PTZ (100 mg/kg)	Occidentalin-1202(n) Occidentalin-1202(s)	Wistar rats	i.c.v. i.c.v.	1.50 (1.46–1.54) μg/animal 0.78 (0.37–1.61) μg/animal
i.p. KA (10 mg/kg)	Occidentalin-1202(s)	Swiss mice	i.p.	1.88 (0.43–8.15) mg/kg

#### Histology

For histological analysis, rats were euthanized by an overdose of sodium thiopental (50 mg/kg; Cristália, Brazil) 2 h after KA injection, transcardially perfused through the left ventricle: first with saline (0.9%) and then with paraformaldehyde (4%, PBS pH 7.4 at 4°C). Brains were immediately removed and placed into formaldehyde (4%, PBS pH 7.4) and kept at 4°C for 24 h. They were then rinsed in 10% and 20% sucrose (PBS pH 7.3) at 4°C for 12 h each. Tissue pieces were embedded in Tissue-Tek, frozen and cut (40 µm) on a cryostat (HM 505 E, Microm, Zeiss). The precise position of the guide cannula and injection site was observed in all animals tested and analysed only the results obtained from animals in which the cannula was presented in the correct place (right lateral ventricle), using as reference the Atlas of Paxinos and Watson (1986). Data from rats with guide cannula tips located outside the right lateral ventricle were not included in analyses.

#### C-fos evaluation

To investigate the role of Occidentalin-1202(s) (3 and 1.5 μg/animal), the C-fos protein was stained on the hippocampal formation slices (dentate gyrus—DG, and the Cornu Ammonis areas CA1 and CA3) in KA-induced seizure in rats (*n* = 3–6). The regions were identified based on the Rat Brain Atlas.^29^ Three slices of each selected region from each animal were two times washed with PBS and then treated with a 10% methanol diluted in a 3% hydrogen peroxide solution for 30 min at room temperature and under constant agitation. Next, brain slices were washed two times with 0.3% Triton X-100 in PBS solution. Non-specific binding was blocked by incubating the slices at room temperature for 30 min in 3% goat serum in PBS. Following the non-specific blockage, the primary polyclonal rabbit anti-c-fos antibody (Sigma–Aldrich), diluted 1:1000 with PBS, was added and incubated at 4°C for 48 h. Slices were then washed with PBS and incubated with goat anti-rabbit IgG-biotinylated secondary antibody (Sinapse Biotecnologia) in a 1:100 PBS dilution for 2 h. Next, slices were washed with PBS and treated with Peroxidase-anti-peroxidase soluble complex (PAP) (Sigma) diluted 1:500 with PBS for 1 h and 30 min at room temperature. Immunoreactions were visualized by exposing the slices for 10 min to a solution containing 0.06% 3.3′-diaminobenzidine diluted in PBS for 10 min at room temperature. A solution of 10% hydrogen peroxide was added to the medium with a 1:1 proportion. Slices were kept with this solution for 10 min at room temperature and then washed with PBS, placed on gelatinized slides, dehydrated in graded alcohol, cleaned in xylene and covered by Entellan (Merck). C-Fos-positive neurons were counted in three areas of DG, CA1 and CA3 (7000 µm3 each area) of every slice using the Leica Application Suite (LAS 4.1.0).

#### Evaluation of motor coordination and determination of the therapeutic index

In order to test the motor function of animals, rats were assayed in the rotarod test (Ugo Basile, Italy), which consisted of a rotating bar (Ø5 cm) driven by a motor (speed: 4 rpm). The day before the execution of the test, rats were trained to maintain their equilibrium on the rotarod. The training consisted of three subsequent 1 min attempts at 4 rpm. On the morning of the test day, rats were again tested on the rotarod and only animals able to maintain their equilibrium on the rod were retained for the experimental procedure. The peptides or saline were administered i.c.v. in a volume of 3 μl 10 min before the execution of the test. The latencies to fall off the rod in the following periods of 15, 30, 45, 60 and 75 min were registered. Moreover, the number of animals falling for two subsequent attempts was used to calculate the doses at which 50% of the animals displayed toxicity (TD_50_). The therapeutic index (TI) was determined as a toxic dose (TD_50_) divided by the effective antiepileptic dose for 50% (ED_50_).

#### Antiepileptic screening by i.p. injection

To evaluate the effect of the peptide Occidentalin-1202(s) by systemic administration, mice were divided into four groups (*n* = 4–5/group), and each animal received the peptide via i.p. (4.00, 2.50 and 1.00 mg/kg) and KA 30 min after peptide (12 mg/kg, via i.p.). After a behavioural assessment, mice were euthanized by an overdose of sodium thiopental (160 mg/kg; Cristália, Brazil).

#### Statistical analysis

Normality tests (Shapiro–Wilk, Kolmogorov–Smirnov, D’Agostino and Pearson) were adopted to determine if data were normally distributed prior to statistical testing. The parametric data were submitted to one-way ANOVA followed by the Tukey post-test, and the nonparametric data (Motor coordination) were submitted to the Kruskal–Wallis test followed by Dunn’s multiple comparisons test. All these data are demonstrated by mean ± standard error of the mean (SEM). We considered significant values in which *P<* 0.05. The method of non-linear sigmoidal was used to calculate ED_50_ and TD_50_ values (with 95% confidence interval). The statistical analysis of the experimental data obtained was performed using the GraphPad Prism® 9.0 software (San Diego, USA).

### Phase 3: Chronic model of epilepsy

#### Neuroprotective and antiepileptic screening in a chronic model of epilepsy

To determine the antiepileptic and neuroprotective effect of the peptide in the chronic model of epilepsy, we induced *status epilepticus* in rats, by intrahippocampal injection of l μl of pilocarpine (2.4 mg/animal), 5–7 days after the surgery procedure. The stereotaxic surgery followed the same protocols described previously, with two guide cannulas implantation: one in the left hippocampus (following the coordinates AP = 6.30 mm, ML = 4.5 mm, DV = 4.5 mm, bregma reference)—pilocarpine administration—and the other in the right ventricle (AP = 0.9 mm, ML = 1.6 mm, and DV = 3.4 mm)—peptide administration. Latency to the onset of SE was then observed, which lasted 3 h. The seizures were stopped by injecting i.p. sodium thiopental (40 mg/kg). Animals that did not present SE were excluded from the study.

This model is used to study mesial temporal lobe epilepsy (MTLE), and it is subdivided into three phases: initial insult, silence phase and chronic phase. Occidentalin-1202(s) was tested in two manners: 10 diary doses administered in the silence phase (neuroprotective effect immediately after SE insult; *n* = 8–12) and 10 diary doses after the chronic phase (antiepileptic effect 15 days after SE insult; *n* = 6–12). Animals were treated with Occidentalin-1202(s) (0.10, 0.05 or 0.01 μg/animal) or saline (SE control). In the first group of experiments (neuroprotective group), we evaluated the effect of Occidentalin-1202(s) on the genesis or development of epilepsy, and in the second group, we identified the antiepileptic effect in the chronic phase.

All animals were filmed for 8 h a day, starting 15 days after the SE insult. The incidence of recurrent seizures was analysed by two evaluators who were unaware of the treatments for each animal (double-blind). The evaluators were trained and had an agreement rate of >95%. The total time of recurrent seizures for each group was quantified to assess the treatment effect in the chronic model induced by pilocarpine.

#### Nissl staining with Cresyl Violet

The pilocarpine-induced SE promotes morphological changes, which may be assessed by classical histological techniques, such as Cresyl Violet Staining (Nissl staining). This technique can be used for qualitative analysis of cell layers organization of the hippocampus, as well as some aspects of cell morphology. In addition, the number of apparently viable cells can be estimated using a quantitative approach. Upon completion of the experiments, animals received an overdose of sodium thiopental, and were intracardially perfused with saline and formaldehyde solutions through the left ventricle. Blood was removed using 0.9% cold saline solution (40 ml to 4°C), followed by 200 ml of 4% formaldehyde (0.5 M phosphate buffer, pH 7.4, 4°C) for 15 min under 50 mmHg pressure. Brain tissues were removed and fixed in 4% formaldehyde solution for 12 h. After this period, brains were placed in 30% sucrose solution (0.1 M phosphate buffer) for 24 h. Brains were then immersed in 2-methyl butane (Sigma), frozen on dry ice for 30 min, covered with Tissue-Tek and then cut in a cryostat (40 slices). Sections were mounted on slides and stained with Nissl to count the viable cells’ ratio and area to be analysed. Regions were selected according to the atlas of Paxinos and Watson (2004).^29^ Cell count was performed by observation under an optical microscope (Olympus BX60). Images were obtained in sequence through a camera. Pictures were then selected to obtain five countable fields. KS400 software (Carl Zeiss Vision, Germany) was used for cell counting of digital images.

### Phase 4: Adverse effects

In order to evaluate undesirable effects induced by Occidentalin-1202(s), healthy rats were treated and subjected to various tests to assess changes in cognitive deficits and loss of motor coordination (ataxia). Groups (*n* = 4–6) were divided into treated groups (0.15, 0.10 and 0.01 µg/animal) and control groups (Saline and Pilocarpine SE).

#### Cognitive deficits

To analyse the presence of cognitive deficits after treatment, rats were tested for spatial navigation in the Morris water maze (MWM). In addition to the experimental groups, control groups were tested: with surgery and injected with saline and a group of animals without surgery. The water maze is made up of a white polyethylene circular pool (140 cm diameter and 50 cm deep) with water (23°C) to a height of 25 cm and added 2 l of milk to prevent the viewing platform. A white platform 9 and 23 cm in height and diameter, respectively, is located in the southeast quadrant. Visual cues were placed on the sides of the water maze. After 10 days of treatment with Occidentalin-1202(s), we performed 6 training sessions per day for 4 consecutive days. The animals were placed in the pool and left for 90 s or until they found the platform. If not found, the animal was removed from the water and placed on the platform. During the training, the animals were kept on the platform to rest for 30 s. The latencies to find it were recorded and used for statistical analysis. On the fourth day after the training trials, the platform was removed, and the rats had to swim for 90 s with no possibility of escape (Probe trial).

#### Motor impairment

In order to test the motor function of animals, rats were assayed in the rotarod test (Ugo Basile, Italy), which consisted of a rotating bar (Ø5 cm) driven by a motor (speed: 4 rpm). The day before the execution of the test, rats were trained to maintain their equilibrium on the rotarod. The training consisted of three subsequent 1 min attempts at 4 rpm. On the morning of the test day, rats were again tested on the rotarod and only animals able to maintain their equilibrium on the rod were retained for the experimental procedure. The peptides or saline were administered i.c.v. in a volume of 3 μl 10 min before the execution of the test. The latencies to fall off the rod in the following periods of 15, 30, 45, 60 and 75 min were registered. Moreover, the number of animals falling for two subsequent attempts was used to calculate the doses at which 50% of the animals displayed toxicity (TD_50_). TI was determined as a toxic dose (TD_50_) divided by the effective antiepileptic dose for 50% (ED_50_).

#### Statistical analysis

The parametric data were submitted to one-way ANOVA followed by the Tukey post-test, and the nonparametric data (Motor coordination) were submitted to the Kruskal–Wallis test followed by Dunn’s multiple comparisons test. Data of the MWM were submitted to two-way ANOVA followed by the Bonferroni post-test. All these data are demonstrated by mean ± SEM. We considered significant values in which *P* < 0.05. The method of non-linear sigmoidal was used to calculate ED_50_ and TD_50_ values (with 95% confidence interval). The statistical analysis of the experimental data obtained was performed using the GraphPad Prism® 9.0 software (San Diego, USA).

### Phase 5: Computational models

#### Molecular dynamics and docking pose evaluation

##### Occidentalin-1202 physiologic conformation

Occidentalin-1202 conformational behaviour was evaluated under hydrophilic and hydrophobic environments. The peptide was created in its linear amino acid sequence (containing the C-terminal amidation) using Discovery Studio software and saved as a PDB file. Using the software VMD, Occidentalin-1202 was inserted in a 56 Å edge water box (16 518 atoms) neutralized with sodium ions (Supplementary Fig. 4A). A molecular dynamic of 30 ns was performed using periodic boundaries, and the secondary structure was analysed by one- and two-dimensional time-related Root Mean Square Deviation (RMSD) (Supplementary Figs 2, 3A and B). The initial water box was modified by adding a 100 × 100 × 210 Å POPC (1-palmitoyl-2-oleoyl-sn-glycero-3-phosphocholine—204 890 atoms) double-layered membrane, thus representing both the hydrophilic and hydrophobic environments (Supplementary Fig. 4B). A molecular dynamic was performed for 30 ns, applying periodic boundaries (Supplementary Figs 10 and 11). Dimerization possibility was also evaluated by inserting another Occidentalin-1202 molecule into the system (Supplementary Fig. 4B).

##### Receptor’s selection for peptide interaction

Since Occidentalin-1202(s) showed a potent antiepileptic performance in the KA-induced acute epileptic model, one hypothesis is that the peptide may interact with kainate receptors. Kainate-agonist receptors were searched in the Protein Data Bank (https://www.rcsb.org/). After filtering for no mutated structures and kainate affinity, ionotropic glutamatergic receptors GluR5 and GluR6 were chosen. This screening gave eight structures, that were superimposed to carry out the alignment of the sequences. A high similarity among the targets was pointed out, with an identity of 84.3% and similarity of 93.5% (Supplementary Fig. 12).

Regarding the crystallographic structures of the receptor, it was possible to verify a good overlap, and a well-preserved position of the connection site between the structures (Supplementary Fig. 13). The average RMSD between all aligned structures was around 1 Å (Supplementary Table 1). Following, two of the best-resolved PDB structures were used to evaluate the kainate binding site, 2XXR (resolution 1.60 Å) and 2XXT (resolution 1.90 Å). Both represent the same GluR6 receptor, but crystallographic structures were obtained with glutamate and kainate in the binding site, respectively. The two PDB structures were aligned and superimposed (RMSD = 0.693 Å), allowing to evaluate kainate ligand domain, showing that both agonists share the same binding site (Supplementary Fig. 14), although glutamate interacts with more residues than kainate (Supplementary Fig. 15).

##### Molecular docking, pose analysis and density functional theory calculations

Docking analyses were performed using GOLD software^30^ (Cambridge Crystallographic Data Center). Water and ligands were removed prior to the study, and the kainate previous position was established as the binding site (a 10 Å radius was used to define the pocket). The system was configured to perform 300 runs and a 200% search efficiency was used in the genetic algorithm. The Occidentalin-1202 molecule used as a ligand was constructed in Discovery Studio as previously informed and exported as a .mol2 file. The docked peptide run that achieved the highest PLP. Fitness score was used to pose (Supplementary Fig. 5) and DFT (density functional theory—Supplementary Fig. 6) analysis in Discovery Studio software.

##### Molecular dynamics

The best pose obtained in the molecular docking was also used to evaluate Occidentalin-1202 interaction with the kainate receptor. The selected PDB structure (2XXT) is a homodimer (Supplementary Fig. 13), so only chain A was used to increase the analysis speed. Using the Discovery Studio software, 2XXT-A and Occidentalin-1202 were included in an orthorhombic 60 A water box equilibrated with sodium and chlorine ions (total 27 322 atoms). Then, the system was minimized and equilibrated to perform the dynamics cascade for 5 ns (2500 frames). The software provided the RMSD, Root Mean Square Fluctuation (RMSF) and radius of gyration calculations. The four frames that presented the lowest energetic interactions are presented in Supplementary Fig. 7.

## Results

As regards the structure of this study, it was divided into five phases, described as follows: Phase 1 concerned the extraction, isolation and purification of Occidentalin-1202(n), followed by the synthesis of an identical analogue peptide, named Occidentalin-1202(s). In Phase 2, we described the effects of both peptides in two acute models of epilepsy—KA and PTZ-induced model of seizures—and measured estimated ED_50_ and TI values, EEG studies and C-fos evaluation. Phase 3 was a compilation of advanced tests performed with Occidentalin-1202(s) only, reporting histopathological features and its performance in the pilocarpine-induced SE. After the determination of the antiepileptic activity of Occidentalin-1202(s), Phase 4 consisted of evaluating its potential adverse effects, after chronic administration, on motor coordination (Rotarod) and cognitive impairment (MWM) tests. Finally, in Phase 5, we proposed a mechanism of action using computational models with kainate receptors.

### Phase 1

#### Peptide discovery

**Figure 1:**
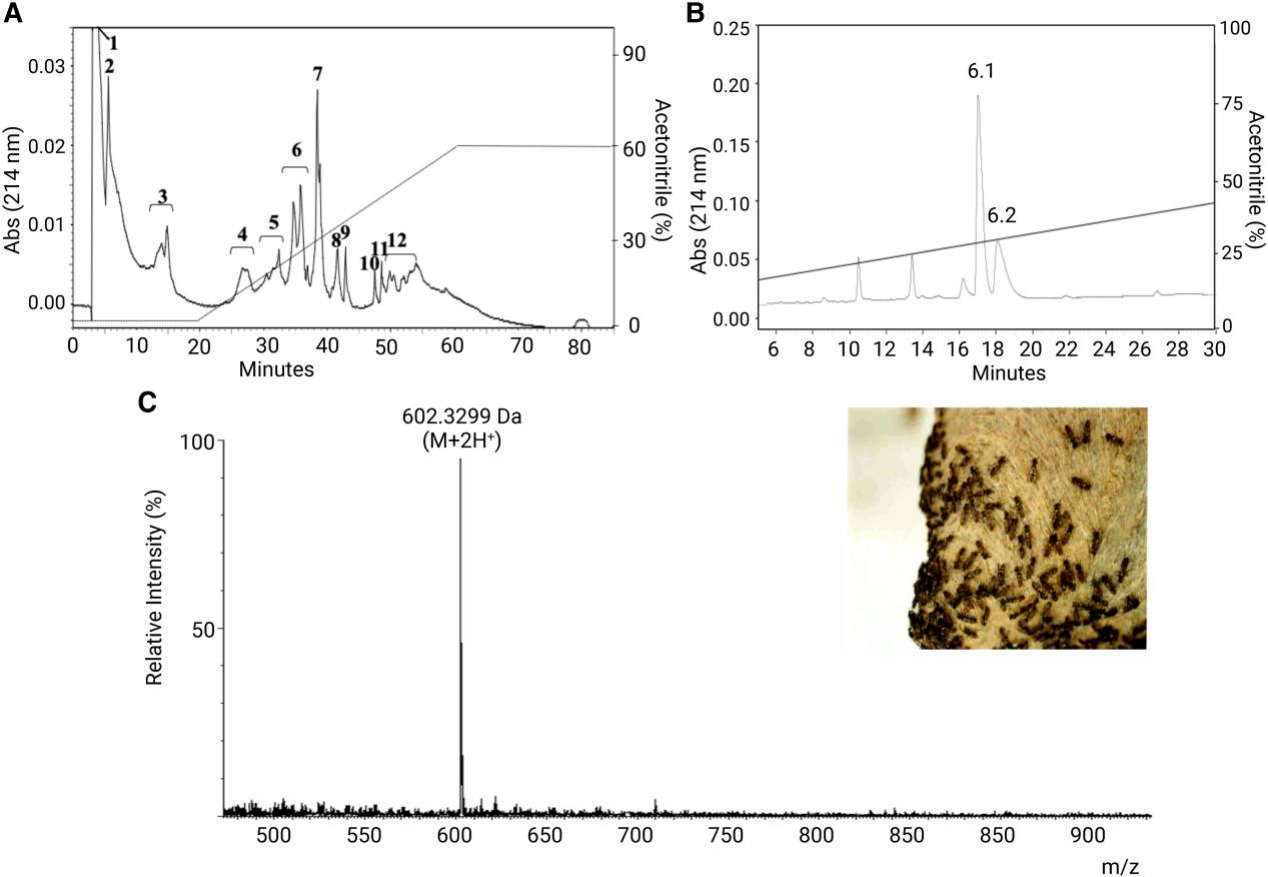
**Extraction, isolation and identification of the natural peptide Occidentalin-1202(n).** (**A**) Chromatographic profile of the venom of the wasp *P. occidentalis* (compounds <3000 Da). We used a reverse-phase column (C18 ODS, 15 μm, 20 × 250 mm) eluted with ACN and TFA 0.07% solution under a flow (2:98) for 20 min, followed by a gradient from 2% up to 60% of ACN for 40 min, closing isocratically (60:40) ACN: TFA 0.07% solution for 20 min. The flow rate was kept at 5 ml/min all the time. The eluted fractions were monitored at 214 nm. The continuous line shows the acetonitrile concentration. (**B**) Second step of isolating fraction 6 by HPLC using a reversed-phase column (C18 ODS Shimadzu 5 μm, 4.6 × 250 mm) eluted with ACN and TFA 0.07% solution through a linear gradient from 10 to 60%, for 40 min with a flow rate of 1.5 ml/min. The continuous line shows the acetonitrile gradient. (**C**) Mass spectrometry ESI/MS high-resolution profile of 6.1 fraction purified by HPLC.

In search of new peptides from wasp venom with neuroactivities, we isolated and sequenced Occidentalin-1202(n) from the crude venom of the Brazilian social wasp, *P. occidentalis*. This species of wasp is endemic to neotropical regions and widely distributed across Latin America.^31^ The first step was separating compounds with molecular weights <3000 Da, fractionated by HPLC. We obtained a chromatographic profile with 12 fractions, in which the neuroactive fraction is represented with an asterisk (Fig. 1A). The mass spectrum (ESI+) of this fraction revealed the presence of two predominant compounds and some contaminants, requiring a new stage of purification.

After the second purification, we detected the separation of compounds that constituted the initial fraction (Fig. 1B). Sub-fraction A (6.1) was analysed by mass spectrometry, and we obtained a pure peptide with *m*/*z* 602 Da ([M + 2H]^2+^), which refers to ionization states of the molecules in the mass spectrometry (Fig. 1C), then named Occidentalin-1202(n).

**Figure 2:**
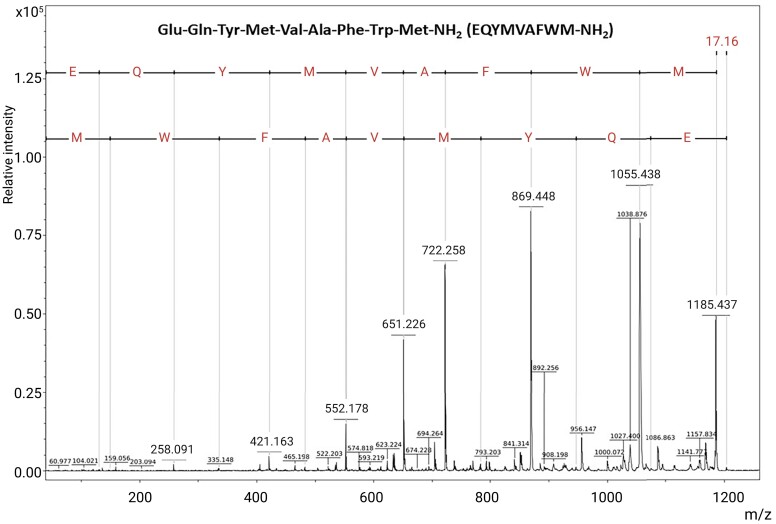
**Determination of the amino acid sequence of the Occidentalin-1202(n).** ESI-MS/MS spectrum of the active peptide, indicating the amino acid sequence at the top of the figure. A single-charged (protonated) molecule was submitted to CID with argon gas at 10–50 eV collision energies.

Based on analyses of the fragmentation diprotonated ion, it was possible to obtain the complete amino acid sequence of the peptide (Fig. 2). The whole series of *b*-ions begins with ion *m*/*z* 129 and follows up with the ion *m*/*z* 258, 421, 552, 651, 722, 869 and 1055. Subtraction of the values of *b*-ions enabled us to obtain the complete sequence of amino acids constituting the peptide, which was confirmed with the identification of *y*-ions (*m*/*z* 149, 335, 482, 553, 652, 783, 946 and 1074), as shown in Fig. 2. For the distinction between glutamine and lysine at Position 2, the peptide was subjected to acetylation. After analysing its mass spectra, we concluded that the residue in Position 2 is the amino acid glutamine (data not shown).

Thus, the Occidentalin-1202(n) complete amino acid residue sequence was identified as **Glu-Gln-Tyr-Met-Val-Ala-Phe-Trp-Met-NH_2_ (EQYMVAFWM-NH_2_)**.

### Phase 2

#### Antiepileptic screening of the peptides in an acute model of seizures

**Figure 3:**
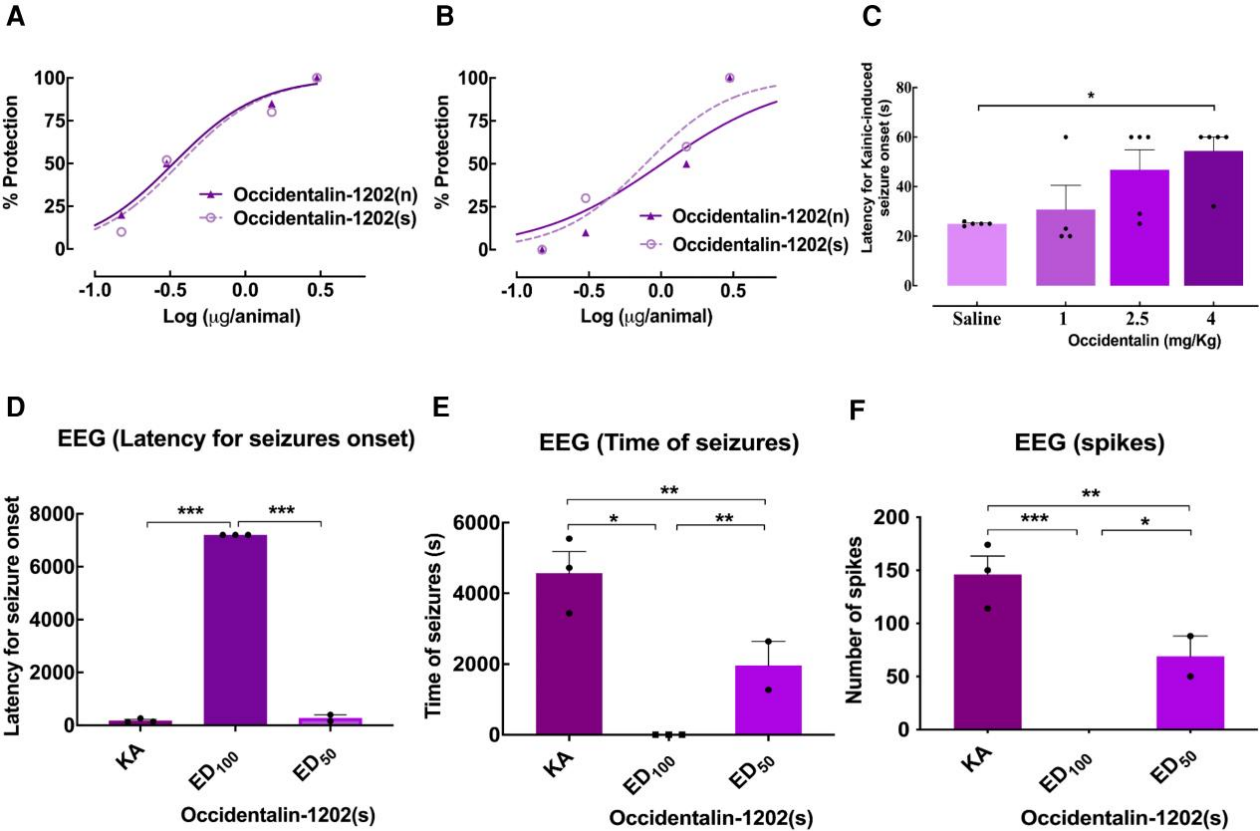
**Occidentalin-1202(n) and Occidentalin-1202(s) (i.c.v. or i.p. via) effect profile against the acute model of seizures induced by convulsant.** Curves of protection obtained by sigmoidal non-linear regression to calculate ED_50_, *n* = 4–6/group, in (**A**) KA (*R*^2^ = 0.9894 and 0.9508, respectively) and (**B**) PTZ-induced acute model of seizure (*R*^2^ = 0.9912 and 0.9536, respectively). The data are presented as % protection calculated by the following formula: (% protection = *N* protected animals/*N* total × 100). (**C**) Effect of the treatment with Synthetic Occidentalin-1202(s) injected by i.p. route against seizures induced by KA (10 mg/kg), *n* = 4–5/group. Histograms represent the latency for kainic-induced seizure onset (s) to score 8 seizures of corresponding values measured from the different experimental groups (Saline, 4, 2.5 and 1 mg/kg of Occidentalin). Analysis of latencies for kainic-induced seizures onset was measured as the time immediately after injection and the onset of score 8 seizures. Data are presented as means of latency for seizure onset ± SEM. Each dot represents one mouse. One-way ANOVA followed by Tukey’s post-test, *P < 0.05 in relation to the saline group. (**D–F**) Representation of the EEG profile of Occidentalin-1202(s) in KA-induced acute model of seizures in rats (*n* = 3). (**D**) Latency for seizure onset. Histograms represent the latency for seizure onset of EEG seizures in seconds evaluated for 2 h after induction. The dose regimen selected was administration of occidentalin-1202(s) Saline (KA), ED_50_ (0.33 µg/animal; *n* = 3) and ED_100_ (3 µg/animal; *n* = 3) by i.c.v. route 10 min prior to KA injections. Data are presented as the mean of latency for seizure onset ± SEM. Each dot represents one mouse. One-way ANOVA followed by Tukey’s post-test; *P < 0.05 and ***P < 0.001 in relation to experimental groups. (**E**) Total time of seizures. Histograms represent the time of seizures in seconds of EEG profile evaluated for 2 h after induction. The dose regimen selected was administration of occidentalin-1202(s) Saline (KA), ED_50_ (0.33 µg/animal; *n* = 3) and ED_100_ (3 µg/animal; *n* = 3) by i.c.v. route 10 min prior to KA injections. Data are presented as the mean of time of seizures ± SEM. Each dot represents one mouse. One-way ANOVA followed by Tukey’s post-test; *P < 0.05 and **P < 0.01 in relation to experimental groups. (**F**) The number of spikes during EEG records. Histograms represent the number of spikes of the EEG profile evaluated for 2 h after induction. The dose regimen selected was administration of occidentalin-1202(s) Saline (KA), ED_50_ (0.33 µg/animal; *n* = 3) and ED_100_ (3 µg/animal; *n* = 3) by i.c.v. route 10 min prior to KA injections. Data are presented as the mean of the number of spikes ± SEM. Each dot represents one mouse. One-way ANOVA followed by Tukey’s post-test; *P < 0.05, **P < 0.01 and ***P < 0.001 in relation to experimental groups. Created with BioRender.com.

We first analysed the peptides’ effects by i.c.v. administrations in rats, using four doses for each peptide (3.00, 1.50, 0.30 and 0.15 μg/animal; *n* = 4–6): Occidentalin-1202(n) and Occidentalin-1202(s) were tested for KA-induced and PTZ-induced seizures. Interestingly, both peptides exerted a dose-dependent anticonvulsant effect in both models. Occidentalin-1202(n), at the lowest dose (0.15 μg/animal), protected 20% of rats from KA-induced seizures, whereas at the dose of 1.50 μg/animal, both Occidentalin-1202(s) and Occidentalin-1202(n) protected 80% and 85% of animals in KA-induced epilepsy, respectively. When the intermediate dose (1.50 μg/animal) was applied to the PTZ seizure model, we observed a protection range of 50–60% between peptides. Additionally, the highest dose (3 μg/animal) exhibited maximum seizure protection (100% of animals protected) in both peptides against the two acute epileptic models (Fig. 3A and B).

We also evaluated the effect of Occidentalin-1202(s) systemic administration (4.00, 2.50 and 1.00 mg/kg; *n* = 4–5/group) in mice. Occidentalin-1202(s) was able to protect against seizures induced by KA after i.p. administration in a dose-dependent manner, indicating that the peptide can cross the blood–brain barrier [*F*(3,15) = 4.422, *P* = 0.0204] (Fig. 3C). Table 1 describes the peptides’ ED_50_ values with confidence intervals calculated for each chemoconvulsant. The ED_50_ values indicate that Occidenalin-1202(n) and Occidentalin-1202(s) were more efficient in the KA-induced seizures, with a lower value and in a decreasing row of efficiency to PTZ.

#### Evaluation of motor coordination and determination of the TI

Alterations in motor coordination and determination of TI were performed with the rotarod apparatus (Ugo Basile, Italy). We evaluated the time that rats spent on the apparatus, starting 1 h after receiving a single i.c.v injection of Occidentalin-1202(s) or Occidentalin-1202(n) (3, 6 and 12 μg/animal; *n* = 5–8). The purpose of this test was to observe whether the peptides would induce effects on neuromuscular coordination. Table 2 indicates the overall values of the time that animals spent on the rotating bar after three attempts. Data are presented as a total number of falls and performance in seconds (mean time ± standard). Interestingly, the lowest and intermediate doses (3 and 6 μg/animal) did not alter motor behaviour in animals, whereas the highest dose (12 μg/animal) increased the latency of fall, possibly affecting rats’ motor coordination. The TD_50_ to alteration in motor coordination values identified were 12.73 and 9.75 μg/animal for and Occidentalin-1202(n) or Occidentalin-1202(s), respectively. Then, the TI values were calculated to assess the safety of the peptides. This index is a comparison of the amount of a therapeutic agent that causes the therapeutic effect to the amount that causes toxicity. The TI values for the acute model of seizures identified were 38,58 and 27,86 for Occidentalin-1202(n) and Occidentalin-1202(s), respectively.

**Table 2:** Effects of Occidentalin-1202(n) and Occidentalin-1202(s) on motor coordination after 45 min of treatment

Administration	Treatment (i.c.v. route)	Performance in seconds (Mean ± S.E.M.)	Number of animals/number of animals that failed in the rotarod
Single dose	Control	60 ± 0	8/0
Single dose	Occidentalin-1202(n) 3 µg/animal	60 ± 0	5/0
Single dose	Occidentalin-1202(n) 6 µg/animal	60 ± 0	5/0
Single dose	Occidentalin-1202(n) 12 µg/animal	**56.8** ± 0.4*	6/6
Single dose	Occidentalin-1202(s) 3 µg/animal	60 ± 0	5/0
Single dose	Occidentalin-1202(s) 6 µg/animal	58.6 ± 1.4	5/1
Single dose	Occidentalin-1202(s) 12 µg/animal	**52.9** ± 1.3***^,**#**^	6/6
Daily dose	Control	60 ± 0	8/0
Daily dose	Occidentalin-1202(s) 0.01 µg/animal	60 ± 0	5/0
Daily dose	Occidentalin-1202(s) 0.10 µg/animal	60 ± 0	5/0
Daily dose	Occidentalin-1202(s) 0.15 µg/animal	**49.6** ± 6.8^*,+,$^	6/2

The Kruskal–Wallis test followed by Dunn’s multiple comparisons test.

Bold text indicates statistical significance: *P < 0.05, ***P < 0.001, in relation to control; ^#^*P* < 0.01 with Occidentalin-1202(s) lowest dose (3 µg/animal); ^+^*P* < 0.05 with Occidentalin-1202(s) lowest dose (0.01 µg/animal); ^$^*P* < 0.01 also with Occidentalin-1202(s) intermediate dose (0.10 µg/animal).

#### EEG evaluation in an acute model of seizures

After establishing the antiepileptic effect of Occidentalin-1202(s) in acute models of epilepsy, we followed this with a more detailed investigation of the peptide profile in the KA-induced model of seizures, using vEEG records (Supplementary Fig. 1). We evaluated three parameters: latency for seizure onset, time of seizures and the number of spikes. Rats were implanted with a head mount and six electrodes bilaterally and in pairs. We used the three-channel tethered system (Pinnacle Technology Inc.), which is capable of recording three EEG traces and, thus, providing observation of distinct areas of the cortex for generalized epileptic seizures (Supplementary Fig. 1A). The dose regimen selected was the administration of ED_50_ (0.33 µg/animal; *n* = 3) and ED_100_ (3 µg/animal; *n* = 3) by i.c.v. route 10 min prior to KA injections. Additionally, we performed a baseline record for 1800 s in all animals before seizure induction to assess their EEG profile (Supplementary Fig. 1B). Rats were then recorded for 2 h after peptide administration and KA injection (Supplementary Fig. 1C–E). Maximal seizure protection was considered as total time analysis with no epileptiform waves (maximum value of 7200 s).

The ED_100_ dose significantly increased the latency for seizure onset and protected against 100% of encephalographic seizures (Fig. 3D). Additionally, both Occidentalin-1202(s) doses extended the latency to seizure (Fig. 3E) and reduced the number of spikes of vEEG records in KA-induced seizures (Fig. 3F). The ED_50_ showed no statistical differences with the control group in latency for seizure onset but was significantly different in decreasing the total time of seizures and the number of spikes (Fig. 3D and E) and reducing the number of spikes in the vEEG records when compared with the KA group. One-way ANOVA followed by Tukey’s post-test revealed that analyses were significant in latency for seizure onset [*F*(2,8) = 5.716, *P* < 0.0001], seizure length [*F*(2,8) = 49.59, *P* = 0.0015] and the number of spikes [*F*(2,8) = 62.41, *P* = 0.0010]. Thus, the peptide Occidentalin-1202(s) exerted a potent antiepileptic effect.

#### Immunohistochemistry (C-Fos)

C-Fos protein is a functional marker involved in several physiological processes and rapidly induced in neurons by different stimulations, such as PTZ-induced kindling, glutamate, kainate, electrical stimulation and norepinephrine.^32^ The purpose of this test was to investigate the role of Occidentalin-1202(s) (1.5 μg/animal, *n* = 5, and 3 μg/animal, *n* = 3) in C-fos expression after KA-induced seizure.

**Figure 4:**
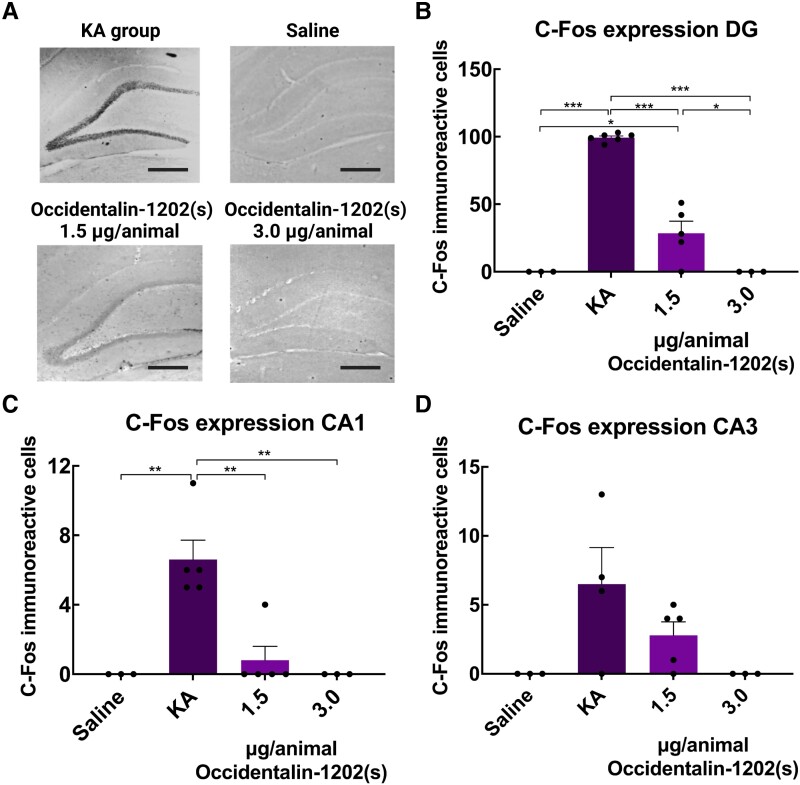
**Effect of Occidentalin-1202(s) in the expression of C-fos immunoreactive cells.** (**A**) Images of C-fos expression of the DG region from the hippocampal formation of animals treated with Occidentalin-1202(s) or receiving 1 µl of saline solution by i.c.v route. Microscopic images obtained from epifluorescence (Leica®), Las 4.1.0. (**B–D**) represent C-fos expression in three regions of the hippocampus (*n* = 3–6): (**B**) DG, (**C**) CA1 and (**D**) CA3 (scale bar: 100 μm). Histograms of the **B–D** figures represent the number of C-fos immunoreactive cells in the dendate gyrus, CA1 and CA3, respectively, evaluated in each experimental group (saline, KA and 3, and 1.5 occidentalin-1202(s) doses). The data are presented as the mean of the number of c-fos immunoreactive cells ± SEM. Each dot represents one slide. One-way ANOVA followed by Tukey’s post-test; *P < 0.05, **P < 0.01 and ***P < 0.001 in relation to different experimental groups. Created with BioRender.com.

We observed that the KA group (*n* = 6) exhibited a marked expression of C-fos protein in the three evaluated regions, whereas the SHAM group (*n* = 6) had no expression of the protein (Fig. 4A–D). Occidentalin-1202(s) (3 μg/animal; *n* = 3) reduced the expression of C-fos, in KA-injected animals, and showed no significant differences when compared with SHAM animals (Fig. 4B–D). Figure 4A illustrates the expression of C-fos protein in the DG region, where it is possible to observe the visual similarities between slices from SHAM and Occidentalin-1202(s) (3 µg/animal) treated animals. One-way ANOVA followed by Tukey’s post-test revealed that analyses were significant in DG C-fos expression [*F*(3,16) = 120.5, *P* < 0.0001], CA1 C-fos expression [*F*(3,15) = 19.6, *P* < 0.0001] and CA3 C-fos expression [*F*(3,14) = 5.364, *P* = 0.0114] (Fig. 4B–D). Therefore, Occidentalin-1202(s) protected cells, and it may exert a potential neuroprotective effect on hippocampal neurons.

### Phase 3

#### Antiepileptic screening of the peptides in a chronic model of epilepsy

**Figure 5:**
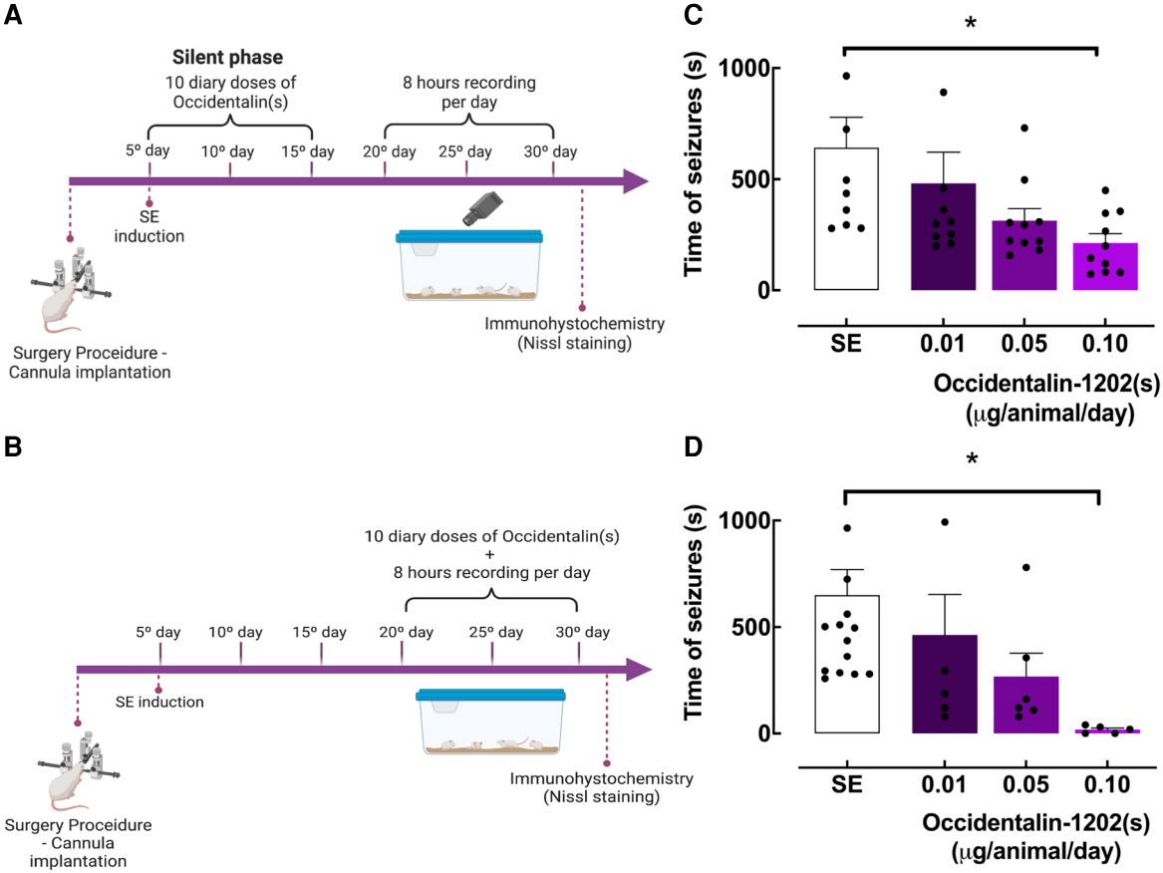
**Occidentalin-1202(s) effect profile against the chronic model of epilepsy.** (**A**) Experimental design for the assessment of neuroprotective effects of Occidentalin-1202(s) injected during silent phase of the pilocarpine-induced recurrent seizure model. (**B**) Experimental design for the assessment of antiepileptic effect of Occidentalin-1202(s) injected during chronic phase. (**C**) Total recurrent seizure time after Occidentalin-1202(s) treatment during the silent phase in seconds. Values represent the means of the total time of recurrent seizures during silent phase treatment measured from the different experimental groups (SE, Occidentalin-1202(s) groups: 0.10, 0.05 and 0.01 µg/animal; *n* = 4–5), with bars denoting SEM. Each dot represents one mouse. All data were subjected to one-way ANOVA followed by Tukey as a post-test. *P < 0.05. (**D**) Total recurrent seizure time after Occidentalin-1202(s) treatment during the chronic phase in seconds. Values represent the means of the total time of recurrent seizures during chronic phase treatment measured from the different experimental groups (SE, Occidentalin-1202(s) groups: 0.10, 0.5 and 0.01 µg/animal; *n* = 4–5), with bars denoting SEM. Each dot represents one mouse. All data were subjected to one-way ANOVA followed by Tukey as a post-test. *P < 0.05. Created with BioRender.com.

We tested Occidentalin-1202(s) in two ways: 10 daily doses administered during the silent phase (neuroprotective effect immediately after SE insult; *n* = 8–12; Fig. 5A) and 10 daily doses after chronic phase (antiepileptic effect 15 days after SE insult; *n* = 5–12; Fig. 5B). Animals were treated with Occidentalin-1202(s) (0.10, 0.05 or 0.01 μg/animal) or saline (SE control).

In the first group of experiments (neuroprotective group), we evaluated the effect of Occidentalin-1202(s) on the genesis or development of epilepsy. In this phase of the study, we observed a decrease in the total recurrent seizure time between the group treated with the highest dose of Occidentalin-1202(s) and the SE group [*F*(3,44) = 1.288, *P* = 0.0276] (Fig. 5C). The value of ED_50_ was 0.023 (0.004–0.095) µg/animal.

In the second group of experiments (antiepileptic group), we observed that the higher dose of Occidentalin-1202(s) reduced the time of seizures in relation to the SE group [*F*(3,44) = 1.344, *P* = 0.0471] (Fig. 5D). The value of ED_50_ was 0.048 (0.011–0.197) µg/animal.

#### Nissl staining

The pilocarpine-induced SE model promotes morphological changes in the CNS of animals, which may be assessed by classical histological techniques, such as Cresyl Violet Staining (Nissl staining). In this test, we analysed the integrity of CA1 pyramid cell layers of pilocarpine-induced SE in rats, after the chronic treatment with Occidentalin-1202(s), counting the proportion of viable cells.

**Figure 6:**
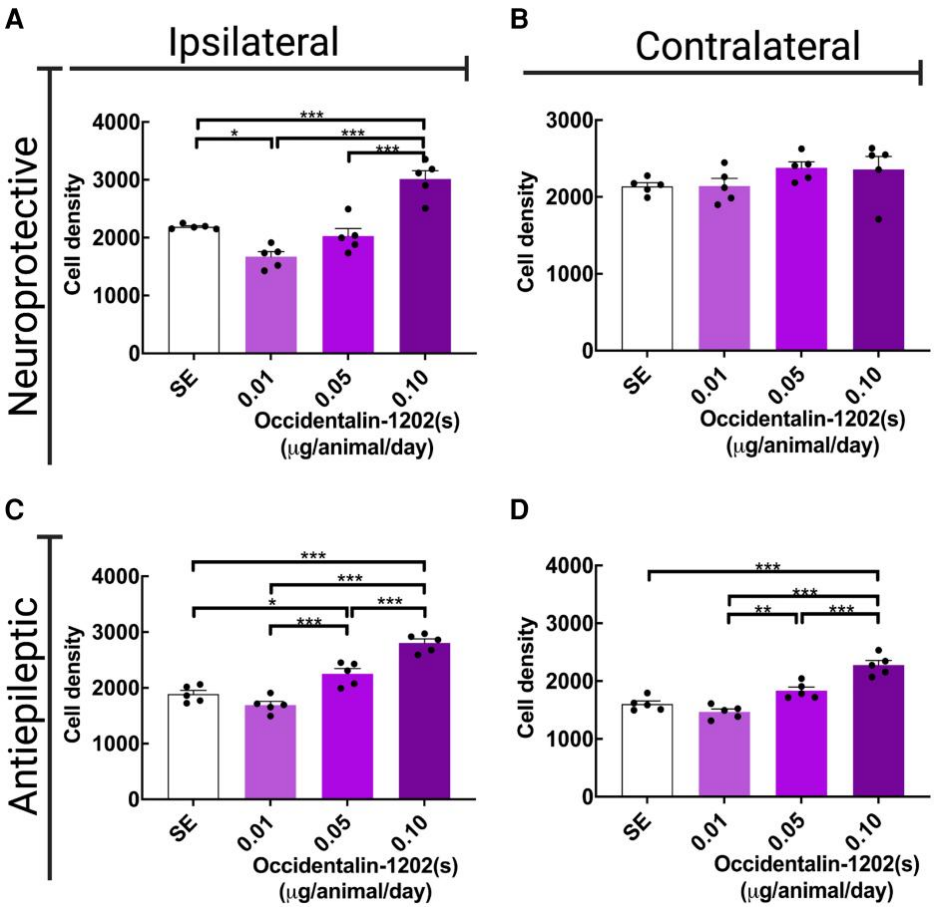
**Occidentalin-1202(s) neuroprotective and antiepileptic effect in counts of CA1 pyramid cell labelled with Nissl.** (**A** and **B**) Cell density of CA1 pyramid cells of hippocampus ipsilateral (**A**), and contralateral sides (**B**), 10 daily doses administered in the silent phase (neuroprotective effect); (**C** and **D**) Cell density of CA1 pyramid cells of hippocampus ipsilateral (**C**), and contralateral sides (**D**), 10 daily doses after chronic phase. Data are presented as the mean of cell density of CA1 layer ± SEM. Histograms show cell density measured from the different groups (SE, Occidentalin-1202(s) groups: 0.10, 0.05 and 0.01 µg/animal; *n* = 4–5). The groups were divided into after silent phase (**A** and **B**) and after chronic (**C and D**) treatments. Each dot represents one slide. One-way ANOVA followed by Tukey’s post-test; *P < 0.05, **P < 0.01 and ***P < 0.001. Created with BioRender.com.

Regarding the neuroprotective group—10 daily doses of Occidentalin-1202(s) administered in the silent phase—the higher dose (0.10 μg/animal) significantly protected neurons in CA1 on the ipsilateral side of the hippocampus (Fig. 6A) [*F*(3,16) = 28.55, *P* < 0.001], whereas on the contralateral side, we did not observe significant differences between the groups (Fig. 6B) [*F*(3,16) = 1.517, *P* = 0.2483].

In the antiepileptic group—10 daily doses of Occidentalin-1202(s) administered in the chronic phase—we observed that the peptide protected neurons on both sides in a dose-dependent manner, with the higher dose (0.10 μg/animal) being highly effective in this model on both sides (Fig. 6C and D). One-way ANOVA followed by Tukey’s post-test revealed that analyses were significant in cell density for the ipsilateral side [*F*(3,16) = 42.13, *P* < 0.001] and contralateral side [*F*(3,16) = 31.88, *P* < 0.001].

### Phase 4

#### Adverse effects

**Figure 7:**
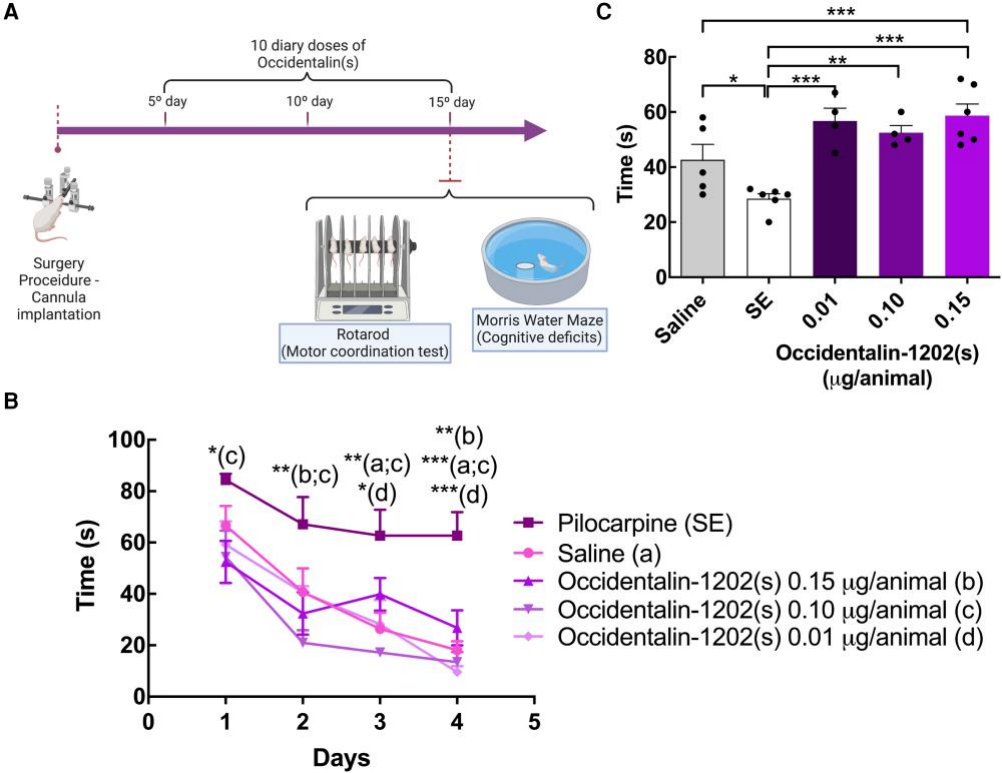
**Evaluation of potential side-effects of Occidentalin-1202 (s).** (**A**) The schematic figure for chronic administration of Occidentalin-1202(s). Created with BioRender.com, USA. (**B**) MWM test; total latency (s) to find the platform for control groups (SE and Saline; *n* = 5–8) and treatment groups (Occidentalin-1202(s) 0.15, 0.10 and 0.01 µg/animal; *n* = 4–5). The data are presented as the mean of latency to find the platform (s) ± SEM measured on each day of the test. Two-way ANOVA followed by the Bonferroni post-test showed significance between the SE group, and Saline and treatment groups; *P < 0.05, **P < 0.01 and ***P < 0.001. (**C**) Pool occupation of rats during the probe trial in the absence of the platform. The data are presented as the mean of pool occupation time (s) ± SEM measured from the different experimental groups: control groups (SE and Saline; *n* = 5–8) and treatment groups (Occidentalin-1202(s) 0.15, 0.1 and 0.01 µg/animal; *n* = 4–5). Each dot represents one mouse. One-way ANOVA followed by the Newman–Keuls multiple comparison tests showed significance between the SE group and treatment groups; *P < 0.05, **P < 0.01 and ***P < 0.001. Created with BioRender.com.

In order to evaluate toxic effects induced by chronic administration of Occidentalin-1202(s), treated rats were subjected to two tests to assess changes in cognitive deficits (MWM) and loss of motor coordination (ataxia) (Fig. 7A).

#### Cognitive deficits

To evaluate the presence of cognitive deficits, rats (*n* = 4–6) were tested for spatial memory and learning in the MWM after chronic treatment (10-day treatment) with Occidentalin-1202(s) (0.15, 0.10 and 0.01 µg/animal; Fig. 7A). In addition to the experimental groups, controls were assessed through Saline (*n* = 8) and SE (*n* = 8) groups.

We observed a significant difference between SE and treatment groups in time spent to find the platform, indicating that SE animals show spatial navigation deficits similar to what has previously been described by others. Interestingly, when compared with Saline groups, none of the Occidentalin-1202(s) doses applied exerted significant differences in spatial navigation (Fig. 7B). Two-way ANOVA showed a significant statistical difference between the SE group, and treatment and Saline groups [*F*(4,23) = 11.12 *P* < 0.001]. Thus, the results indicate that Occidentalin-1202(s) did not alter cognitive performance, and animals were able to learn the platform’s localization.

On the fourth day after the training trials, the platform was removed, and the rats had to swim for 90 s with no possibility of escape (Probe trial). In the Probe trial, all treatments and Saline groups, in contrast to the SE group, spent more time in the quadrant where the platform was located during training when compared with the other quadrants (Fig. 7C). One-way ANOVA showed a significant statistical difference between the SE group and treatment groups [*F*(5,29) = 7.135, *P* < 0.05].

#### Motor impairment

Some anticonvulsants are known to induce adverse motor impairment with chronic use. To address possible motor alterations, we performed the rotarod test after 10 days of Occidentalin-1202(s) treatment. Our results show that only the highest dose affected motor coordination, whereas the lowest and intermediate doses did not alter motor behaviour in animals (Table 2). The estimated value of TD_50_ was 0.32 (0.11–2.83) μg/animal, and the TI values to the chronic model of seizures identified were 13.9 and 6.7 for Occidentalin-1202(s) in neuroprotective and antiepileptic groups, respectively.

### Phase 5

#### Computational models

##### Peptide conformation in the hydrophilic and hydrophobic environments and evaluation of dimerization capacity

In the water box, the peptide showed a tendency to close (Supplementary Fig. 2), probably to protect the hydrophobic side chains. In the POPC bilayer media, the peptide presented a more linear tendency. Glutamic acid, the first residue, provides a negative charge to the peptide, whereas the C-terminal amidation provides a positive charge. The amidation cap seems to be important for the activity of the molecule.^33,34^ In addition, Occidentalin-1202 spent more time in its more defined secondary conformation (Supplementary Fig. 3C).

Dimerization capacity was evaluated to understand the possibility of the reaction between two peptides. Under the established conditions, the peptides did not bond (Supplementary Fig. 4B). An attractive force between the peptides was introduced, strong enough to quickly move each peptide closer (only 10 frames—results not shown). However, no dimer formation was observed.

##### Molecular docking, pose and DFT analysis

**Figure 8:**
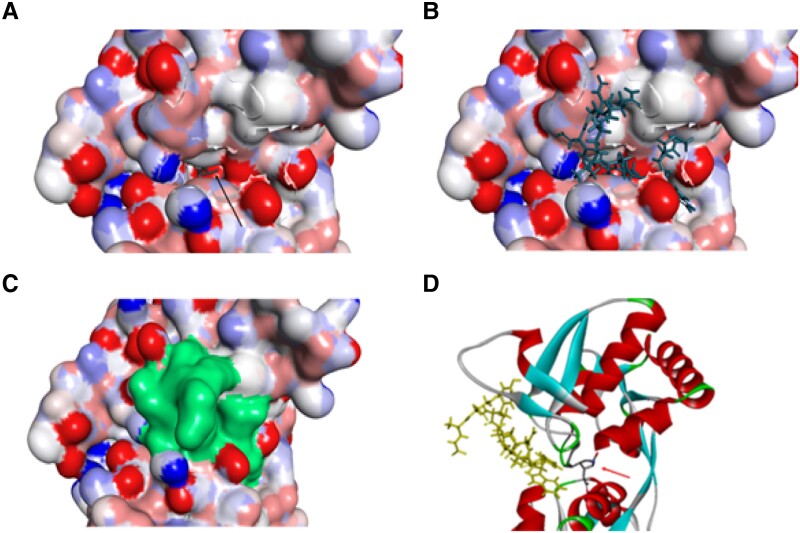
**Glutamate/Kainate receptor (GluR6) binding site.** (**A**) Kainate deep in the pocket (arrow indicates the position). (**B**) Occidentalin-1202 blocking the entrance of the receptor. (**C**) Occidentalin-1202 highlighted the surface, indicating the total blockade of the binding pocket. (**D**) Occidentalin-1202 (yellow) does not occupy the same space as the neurotransmitter kainate (arrow indicates the position) in GluR6 receptor.

The docking procedure was performed 300 times (300 runs), and the best pose obtained presented a PLP. Fitness score of 99.4608. The pose presented the peptide blocking the kainate/glutamate entrance of the binding site (Fig. 8A–C). The last triad of amino acids (Phe-Trp-Met-NH2) is responsible for most of the interactions with the receptor. In this analysis, Occidentalin-1202 appears to fit as a ‘belt’ in front of the binding pocket. The antepenultimate amino acid of the peptide, a phenylalanine residue, appeared closing the pocket entrance through a benzene ring interaction with Tyr488 (pi–pi) and Val685 (pi–alkyl) residues (Supplementary Fig. 5). This ring closing the pocket entrance does not interact with most of the residues of the kainate binding site but seems to prevent kainate from entering the site. Figure 8D is a plot of the receptor and both kainate and Occidentalin-1202, showing that the peptide seems to block the neurotransmitter entrance.

DFT, Highest Occupied Molecular Orbital (HOMO) and Lowest Unoccupied Molecular Orbital (LUMO) analyses (Supplementary Fig. 6) provided results that allow it to be deducted that the best docked pose may be associated with a chemical softness of the molecule (HOMO–LUMO gap = 0.832 eV), which means that this peptide’s pose could be prone to interact with the receptor.^35^

##### Molecular dynamics

The results obtained show that, at least in the observed 5 ns, the system is relatively stable,^36^ with a Total System Energy gap of 1365.7 kcal/mol, RMSD variation of 1.56 Å after equilibration and a Radius of Gyration delta of only 0.5437 Å.

The molecular dynamics provided more information about Occidentalin-1202 interaction with the GluR6 receptor than was possible to evaluate after docking analysis. The most favourable frames (i.e. those with the lowest Total System Energy—Supplementary Fig. 7) showed that the peptide was able to interact weakly with some of the receptor residues in the kainate/glutamate pocket (mainly TYR488, ALA518, ARG523 and ALA689), thus providing a further blocking interaction. RMSF values indicate that most of the least fluctuating residues of the protein were close to or within the Occidentalin-1202 binding site (Supplementary Fig. 8). That shows the possible stabilization of the peptide interaction (Supplementary Fig. 9) pocket when bound.

## Discussion

### Phase 1

#### Peptide discovery

In the present work, our research group isolated and characterized a novel natural peptide, named Occidentalin-1202(n), with potential antiseizure effects, when administered both intracerebrally and systemically. We also described the noteworthy activity of a synthetic analogue peptide, named Occidentalin-1202(s), comparing both compounds in the same convulsant-induced acute seizure models, and determining the effective dose (ED_50_ and ED_100_). The amino acid sequence of the natural Occidentalin-1202(n) was determined, composed of nine amino acid residues: Glu-Gln-Tyr-Met-Val-Ala-Phe-Trp-Met-NH_2_. This peptide has six amino acids with nonpolar groups, two amino acids with non-charged polar groups (Tyr and Gln) and a negatively charged amino acid (Glu). Reviews and comparisons with data in the literature and Protein Data Bank.^37^ (http://www.rcsb.org) did not reveal peptides with sequences similar to Occidentalin-1202(n).

### Phase 2

Indeed, the results of this study demonstrated that the peptides Occidentalin-1202(n) and Occidentalin-1202(s) can cross the BBB and induce a dose-dependent anticonvulsant effect, evaluated after induction of seizures by two chemical-convulsant models, KA and PTZ, at doses compatible with the proposed therapeutics. Interestingly, both peptides were more effective in blocking seizures induced by KA, with the ED_50_ 2–4 times lower than that observed against PTZ. Additionally, after determining the ED_50_ of the two peptides, we evaluated the antiseizure profile of the synthetic analogue through EEG records in the KA-induced seizure model. The results supported the Occidentalin-1202(s) anticonvulsant effect, with the ED_100_ fully protecting animals against KA-induced seizures. In contrast, despite the reduction in the total time of seizure and the number of spikes, the ED_50_ was ineffective in covering latency for seizure onset.

Previous works have demonstrated the unique ability of naturally occurring peptides, isolated from animal venom, to interact with intrinsic physiological processes in cells.^6,38,39^ In fact, peptides extracted from the venom of the *Conus* genus have been reported to have an interestingly anticonvulsant and neuroprotective effect.^40–42^ When a comparison was made between Occidentalin-1202(n) and the anticonvulsant peptide, Conantokin-R, isolated from the venom of *Conus radiatus*, it was found that the peptide isolated in the present study (ED_50_ = 1.50 µg/rat or 1.25 nmol/rat) is about 12.5 times less active than Conantokin-R (ED_50_ = 0.10 nmol/rat) in the PTZ-induced model of seizures.^42^ Although Occidentalin-1202(n) had a lower activity than Conotokin-R, comparisons with the peptide Ppnp7 (ED_50_ = 2.70 nmol/rat), isolated from the venom of the social wasp *Polybia paulista*, showed that Occidentalin-1202(n) is about 2.2 times more active, also in the PTZ-induced model of seizures.^43^

In turn, the anticonvulsant activity in seizures, induced by subcutaneous injection of PTZ, is a way to identify compounds that are effective in the treatment of myoclonic or absence seizures. PTZ and KA are the model compounds most used to search for anticonvulsants.^44^ PTZ is a non-competitive antagonist of GABA_A_ receptors and causes clonic and generalized tonic-clonic seizures when injected systemically.^45^ Among the conventional AEDs, ethosuximide, valproic acid, phenobarbital and benzodiazepines are effective in blocking seizures induced by PTZ, whereas carbamazepine and phenytoin are ineffective or pro-convulsant.^46^ The excitatory neurotransmitter in mammals is mediated primarily by L-glutamate.^47,48^ This neurotransmitter acts on three types of ionotropic receptors: NMDA, AMPA and kainate.^48^ KA is an agonist for glutamate receptors of kainate/AMPA types and when injected in i.c.v route, it can cause tonic-clonic seizures.^46^ Seizures induced by this compound were first proposed by Ben-Ari (1985) as a model of particular relevance in the selection of anticonvulsants that are effective against complex partial seizures and for the treatment of temporal lobe epilepsy in humans.^49,50^ Moreover, some AEDs are ineffective at blocking seizures induced by KA, especially those presenting, as their primary mechanism, the blockage of voltage-dependent sodium channels, such as carbamazepine and phenytoin.^46,51–53^ Numerous data suggest that drugs that act on GABAergic transmission or antagonists of L-glu-type AMPA/kainate are effective in animal models undergoing KA-induced seizures.^54–57^

### Phase 3

Our results are very promising in the chronic model of pilocarpine, which highlights the use of the peptide in the treatment of MTLE. MTLE is the main epileptic disorder in humans and is characterized by the recurrence of disordered activation of areas of the brain limbic system, with high refractoriness (>75% of patients without effective seizure control; for review see Lévesque *et al.*^58^). In this epilepsy, memory deficits are observed, associated with hippocampal sclerosis and damage to various structures of the hippocampal formation resulting from epileptic seizures, as this region plays a role in the acquisition and consolidation of memories.^59,60^ The chronic animal model of MTLE chemically induced by pilocarpine, by mimicking the brain damage of patients with epilepsy, leads to a progression of histopathological and behavioural changes, and also reproduces the cognitive problems associated with this condition.^61^ Our data show that Occidentalin-1202(s) is effective in the control of recurrent seizures induced by pilocarpine status when applied in both the silent (epileptogenic) phase and the post-onset phase of recurrent seizures in the chronic period. In this model, protracted SE induced brain damage, including in the hippocampus formation,^62,63^ which was analysed by us.

We also evaluated the effect of Occidentalin-1202(s) on C-Fos expression to address possible inhibition of hyperexcitability of the brain. Together with members of the Jun family, C-Fos dimerizes to compose a transcriptional factor, activator protein-1 (AP1), which modulates the long-term cellular phenotypical process. The C-fos activation can be differently stimulated by neurotropic factors, neurotransmitters, chemoconvulsants, depolarization and an increase in Ca^2+^ influx and elevation of intracellular/intranuclear Ca^2+^.^32,64^ In fact, C-fos is widely used as a functional marker to identify activated neurons and extended circuitry. It has been established, *in vivo*, that minutes after acute stimulation, there is an increase in C-fos mRNA in different brain regions, reaching a peak at 30–60 min, followed by a gradual decline over 4–6 h (for review see Kovács^64^). In the present study, we identified an increase in C-fos expression 2 h after KA injection. This is similar to other studies that have shown increases in the expression of C-fos in the hippocampus after the induction of epileptic seizures by different chemical epileptics.^10,65^ Interestingly, slices from animals treated with Occidentalin-1202(s) showed a significant reduction of C-fos expression in the three regions of the hippocampal formation (DG, CA1 and CA3) with both applied doses. Since excitotoxicity is considered an important event in epilepsy, which can unleash toxicological features into the neurons, Occidentalin-1202(s) may have prevented the C-fos expression trigger factors, thus exerting a potential neuroprotective effect on neurons from the hippocampal region.

### Phase 4

According to the World Health Organization, the adverse effect of a drug can be defined as: ‘harmful and unintended response generated after the use of drugs in doses that are normally used for prophylaxis, diagnosis, disease therapy, or for the modification of a biological function’.^66^ Regarding the CNS, some of the effects observed may be cognitive deficits, such as memory, and motor changes (ataxia and sedation).^67^ People with epilepsy already have a higher risk of cognitive impairment,^68^ and these disorders can occur as a result of brain damage, communication due to epileptic seizures or because of adverse effects with which AEDs are commonly associated.^69^

In the MWM test, the doses of the peptide Occidentalin-1202(s) did not alter the cognitive capacity of the animals, matching the healthy group. This test is widely used to assess memory and learning deficits.^70^ The importance of studying tests like this in the prospect of a possible new antiepileptic compound is due to the fact that several usual AEDs such as benzodiazepines are often related to memory deficits.^71^ A more effective therapy would ideally be able to suppress epileptic seizures without the cognitive problems. Several antiepileptics have already been tested in the MWM using animal models of induced epilepsy. For example, Valproic acid showed a dose-dependent effect in the worsening of the MWM performance of rats after electrical induction of epilepsy,^72^ and other studies have already demonstrated its effect on the decrease in spatial memory.^73^ Phenobarbital and topiramate worsened MWM performance in rats after electrical induction of epilepsy;^72^ indeed, topiramate is one of the AEDs shown to cause more cognitive deficits, usually affecting memory.^74^

The Rotarod test is widely used to study the toxicity of potential AEDs, allowing the observation of acute difficulties in terms of motor coordination and balance, in addition to being commonly used to quantify TI.^75,76^ The inability of the experimental animal to continuously balance itself on a rotating bar, for a certain period, is used as an indication of motor impairment,^77^ being listed, for example, ataxia reported in human patients.^78^

In the present study, the lowest and intermediate dose of the peptide Occidentalin-1202 did not alter the animals’ motor coordination, being equal to the length of stay of healthy animals. It does not cause adverse effects that are normally associated with the use of AEDs, such as sedation and ataxia. Moreover, an important parameter to be analysed in the clinical use of new AEDs is the TI, expressing the margin between antiepileptic and adverse effects. In our study, we identified a TI around 30 to the acute model of seizures and 6–14 to the chronic model. These values demonstrate the safety of the peptide, since the most used AEDs have a TI of around 2.^79^

### Phase 5

Interestingly, after computational analyses to evaluate possible interactions between Occidentalin-1202(s) and receptor GluR6, a kainate receptor selected for the experiments, we observed a blockage of the receptor’s action site. The results showed that Occidentalin-1202(s) may bind directly to the receptor’s pocket entrance, strongly interacting with amino acid residues around the pocket and performing weak interactions with some of the binding site residues, avoiding the kainate/glutamate inlet. Therefore, we suggest that our peptide may block the kainate receptor, preventing kainate/glutamate binding and, thus, hindering neuron activation and potentially protecting neurons from excitotoxicity. In recent years, studies have investigated the efficacy of glutamatergic antagonist drugs for the treatment of patients with drug-resistant epilepsy.^58^ Among the neuroactive drugs currently available, treatment with ketamine resulted in a decrease in the number of recurrent seizures in patients with refractory seizures.^80^ Currently, the use of ketamine as a first-line antiseizure agent is ongoing in a clinical phase study 3 (ClinicalTrials.gov NCT03115489).

## Supplementary Material

fcad016_Supplementary_Data

## Data Availability

The authors confirm that the data supporting the findings of this study are available within the article and its Supplementary material.

## Abbreviations

ACN =: acetonitrile
AED =: antiepileptic drug
AP =: anteroposterior
CNS =: Central Nervous System
DV =: dorsal–ventral
HOMO =: Highest Occupied Molecular Orbital
HPLC =: High-performance liquid chromatography
i.c.v. =: intracerebroventricular
KA =: kainic acid
LUMO =: Lowest Unoccupied Molecular Orbital
ML =: mesolateral
MTLE =: mesial temporal lobe epilepsy
MWM =: Morris water maze
PAP =: Peroxidase-anti-peroxidase soluble complex
PBS =: phosphate-buffered saline
PTZ =: pentylenetetrazole
RMSD =: Root Mean Square Deviation
RMSD =: Root Mean Square Fluctuation
SE =: status epilepticus
TFA =: trifluoroacetic acid
TI =: therapeutic index
vEEG =: video-electroencephalography
