# Supplementary material for: A new class of peptides from wasp venom: a pathway to antiepileptic/neuroprotective drugs
Source: Brain Commun. 2023 Feb 17;5(1):fcad016. doi: 10.1093/braincomms/fcad016 (PMC9945850; doi:10.1093/braincomms/fcad016)
Supplement: fcad016_Supplementary_Data [file fcad016_supplementary_data.pdf]

Supplementary material

Supplementary Table 1. RMSD from the reference structure (PDB 1TT1) was obtained from the structural alignment of the eight kainate receptor structure sequences obtained from the PDB database.

PDB	RMSD (Å)	Residue numbers
2XXT	0,7426	216
3C31	1,0070	216
3C32	1,0005	216
3C33	1,0284	216
3C34	1,0303	216
3C35	1,0355	216
3C36	1,0155	216

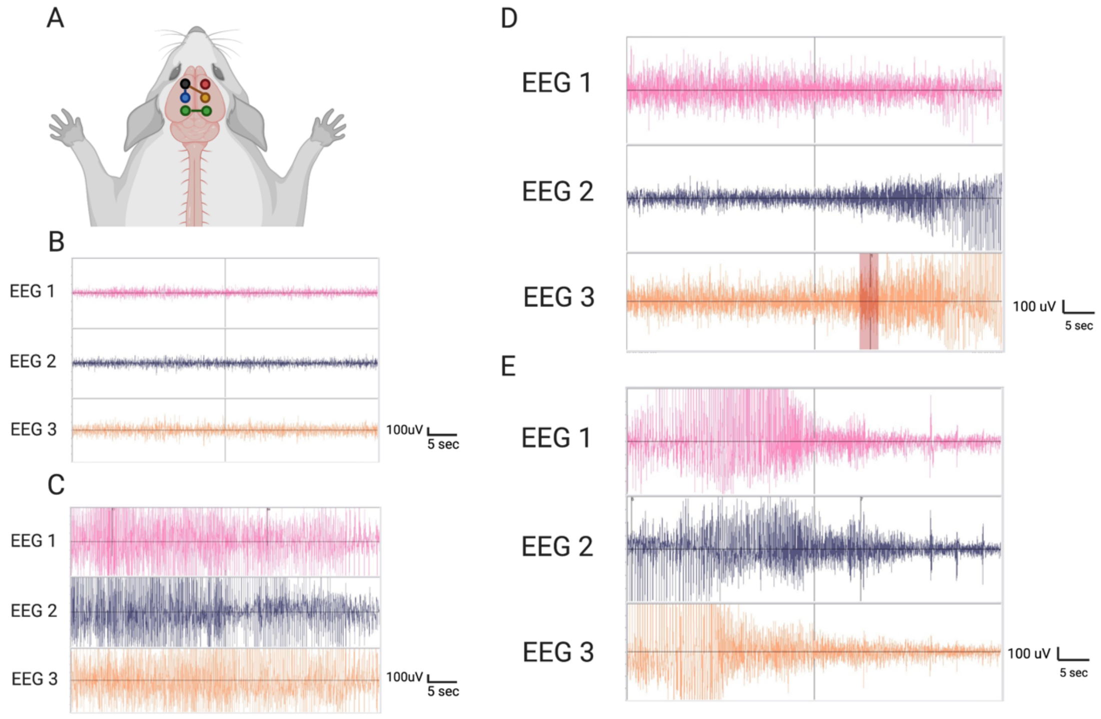

Supplementary Figure 1: (A) Three-Channel system, standard configuration (Pinnacle technology Inc, EEG/EMG systems brochure). The third channel (EEG 3) is independent and was responsible for recording the lateral parietal association cortex (occipital point, dots in green). The second channel (EEG 2) is connected to the reference point (dot in black), and records signals from the right somatosensory cortex (right parietal point, dot in yellow). The first channel (EEG 1) registers signals from the left somatosensory cortex (left parietal point, dot in blue) and is also connected to the reference point. (B) Basal line for EEG record. (C) EEG record of animal with generalized seizure from KA group. (D) EEG record of animal treated with ED_{50} Occidentalin-1202(s), seizure onset. (E) EEG record of animal treated with ED_{50} Occidentalin-1202(s), end of seizure. Created with BioRender.com

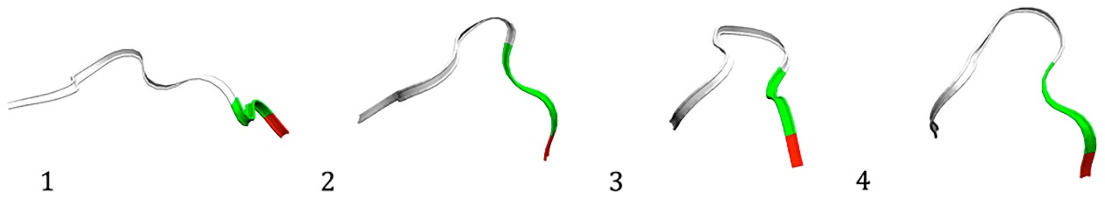

Supplementary Figure 2: Four representative and original conformations of the peptide Occidentalin in molecular dynamics of 30 ns in hydrophilic environment (water box).

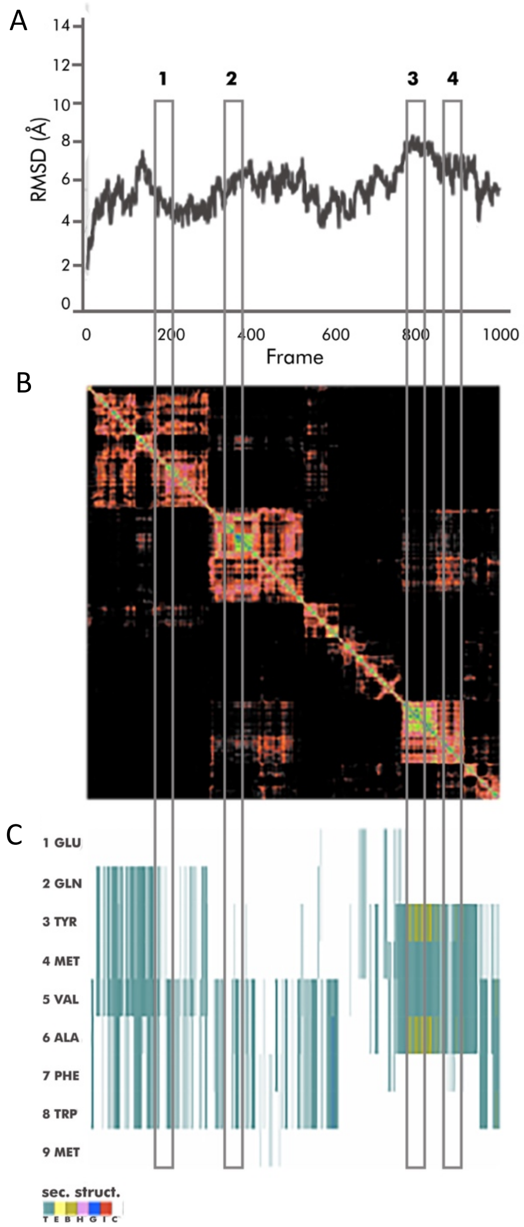

Supplementary Figure 3: Analysis by one-dimensional RMSD (A), two-dimensional RMSD (B) and analysis of secondary structures with the time of the peptide in a hydrophilic environment (C). Four conformations were more representative and original during the dynamics of 30 ns. In the legend, T: turn E: extended conformation; B: bridge; H: alpha-helix; G: helix 3-10; I: pi hélix; and C: coil.

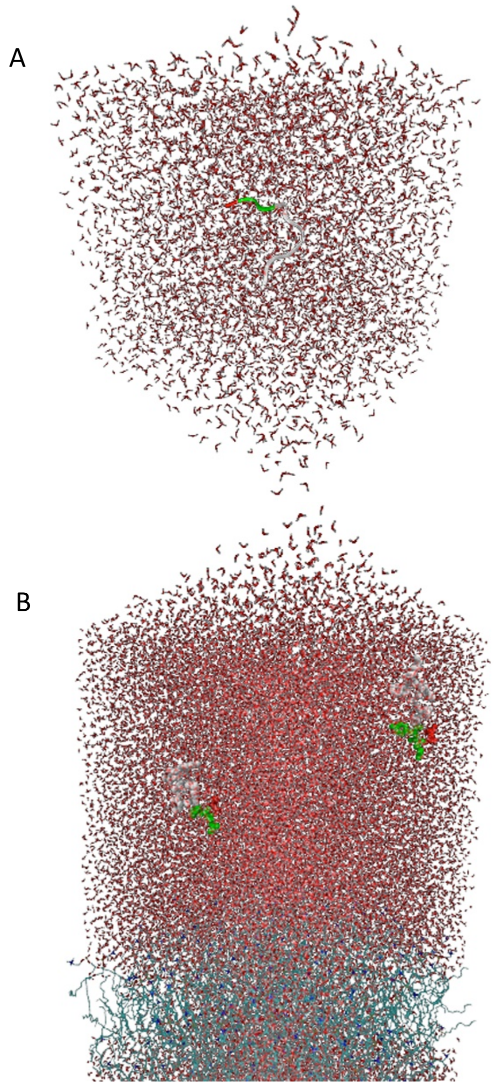

Supplementary Figure 4: (A) Cubic water tank, with 56 Å of edge, peptide centralized, and balanced system (total of 16518 atoms); (B) Water tank with the patch of bilayer lipid, 100 x 100 x 210 Å of edge, and a total of 204,890 atoms.

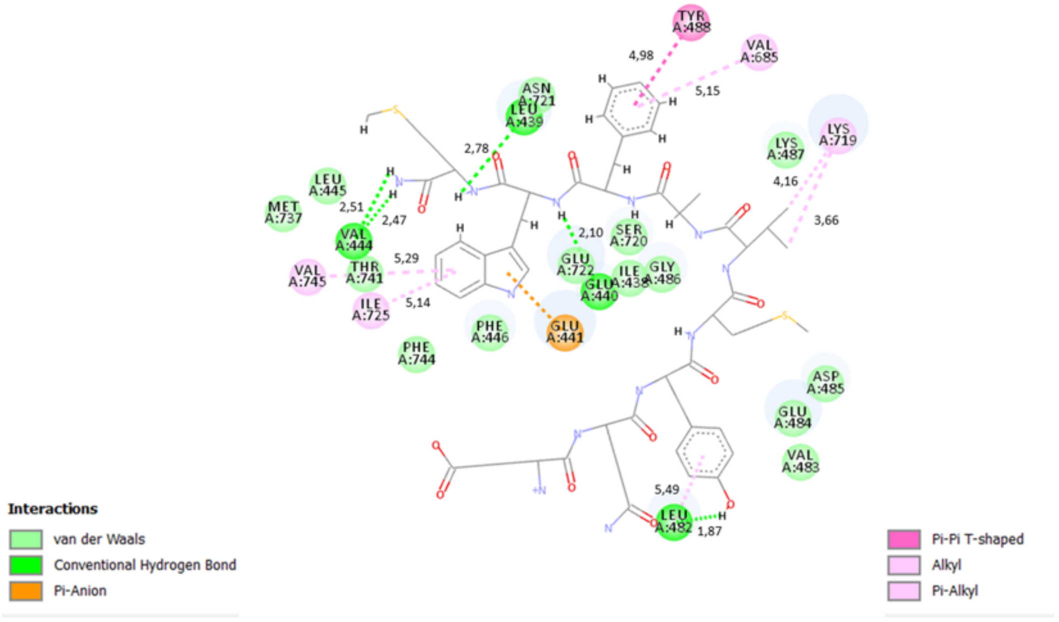

Supplementary Figure 5: Best docking pose interaction analysis. Interaction types vary according to the legend indications. Distances (Å) are shown close to the dashed lines.

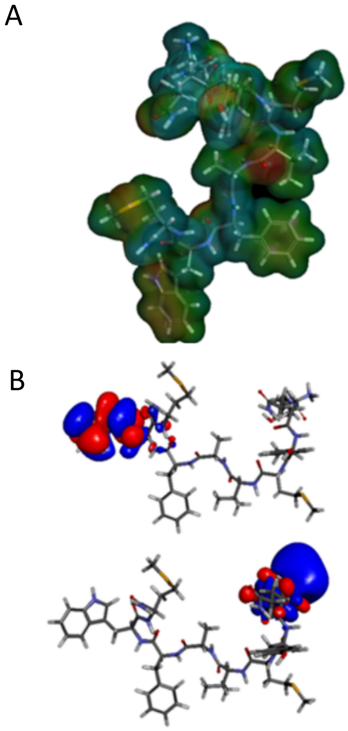

Supplementary Figure 6: (A) Electron density distribution in Occidentalin-1202 best pose obtained in docking process. Red means higher electron density, blue means lower electron density. (B) HOMO and LUMO of Occidentalin-1202.

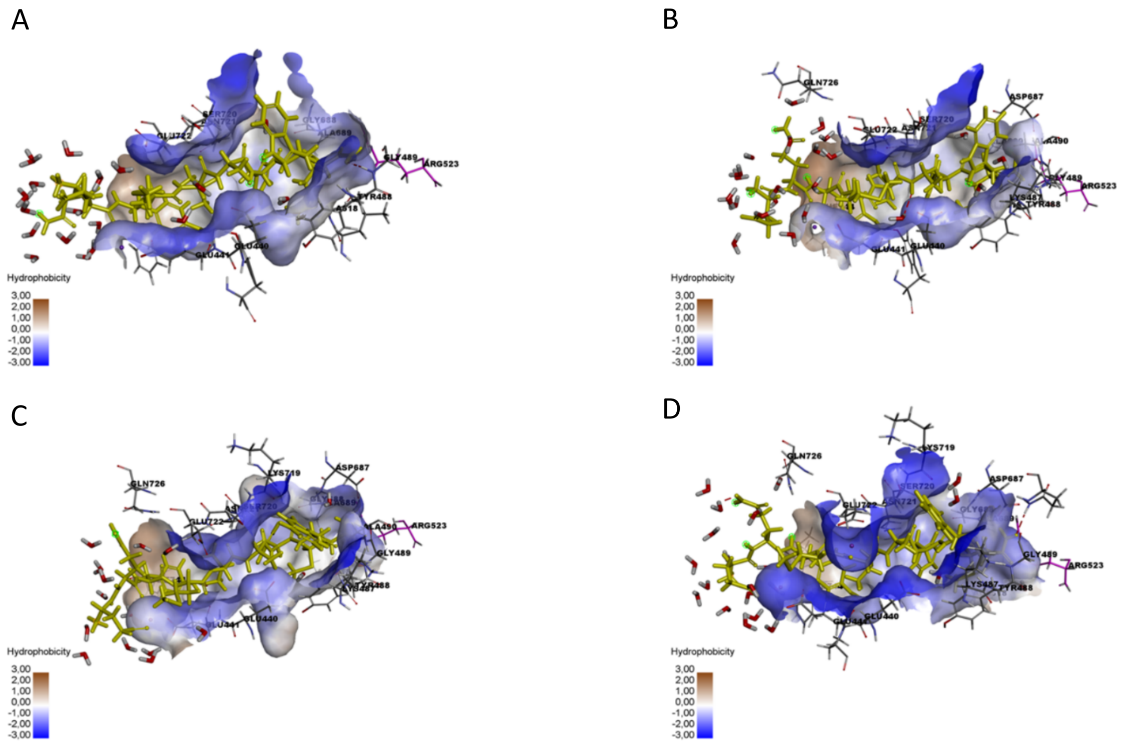

Supplementary Figure 7: Frames 1868 (A), 2378 (B), 4820 (C) and 5096 (D), representing the 4 least energetic conformations of Occidentalin-1202 interacting with GluR6 receptor. PDB: 2XXT.

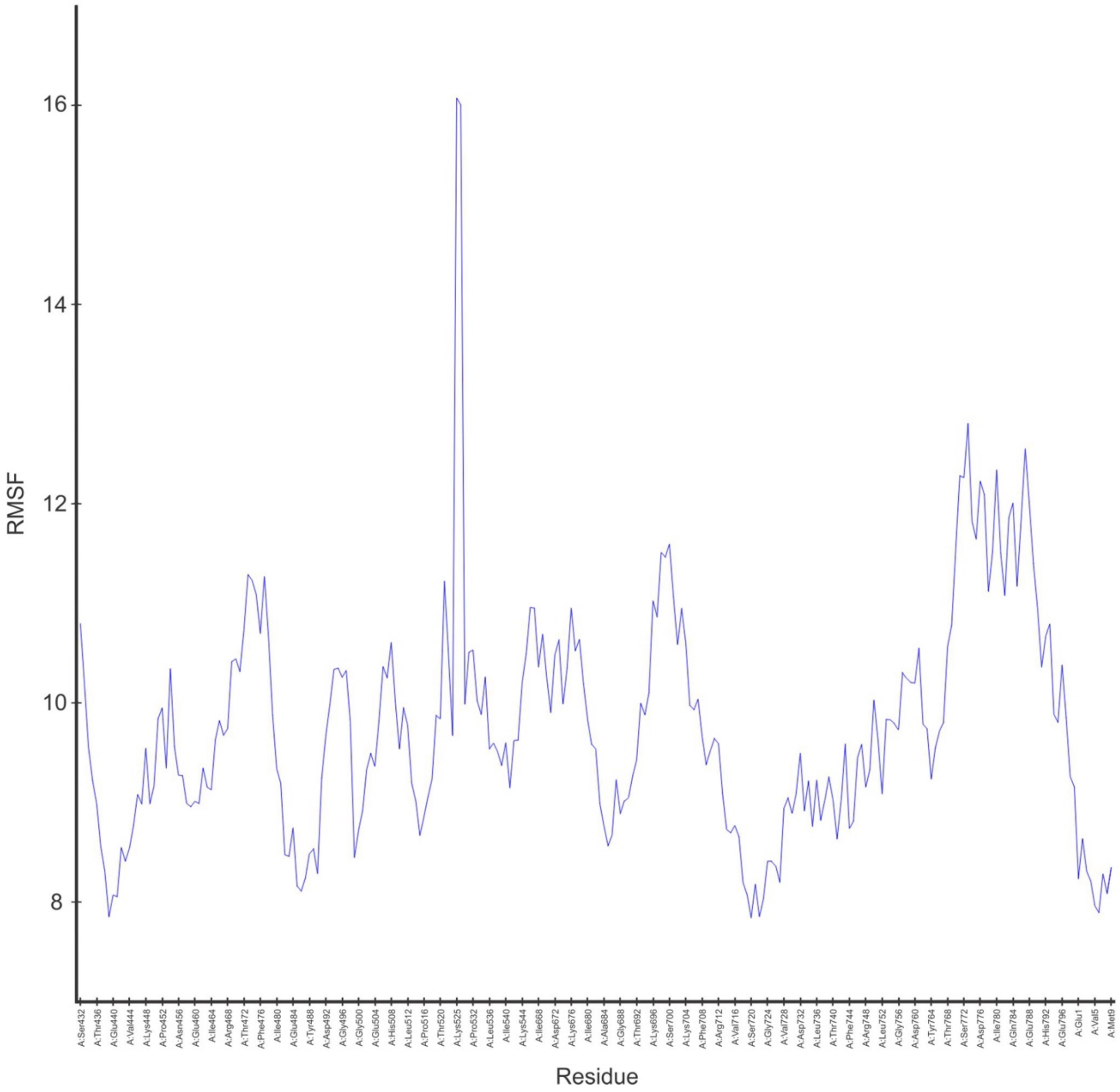

Supplementary Figure 8: Root-Mean-Square Fluctuation (RMSF) during the 5ns Occidentalin-1202 molecular dynamics. GluR6 residues (PDB: 2XXT) are plotted on the x-axis.

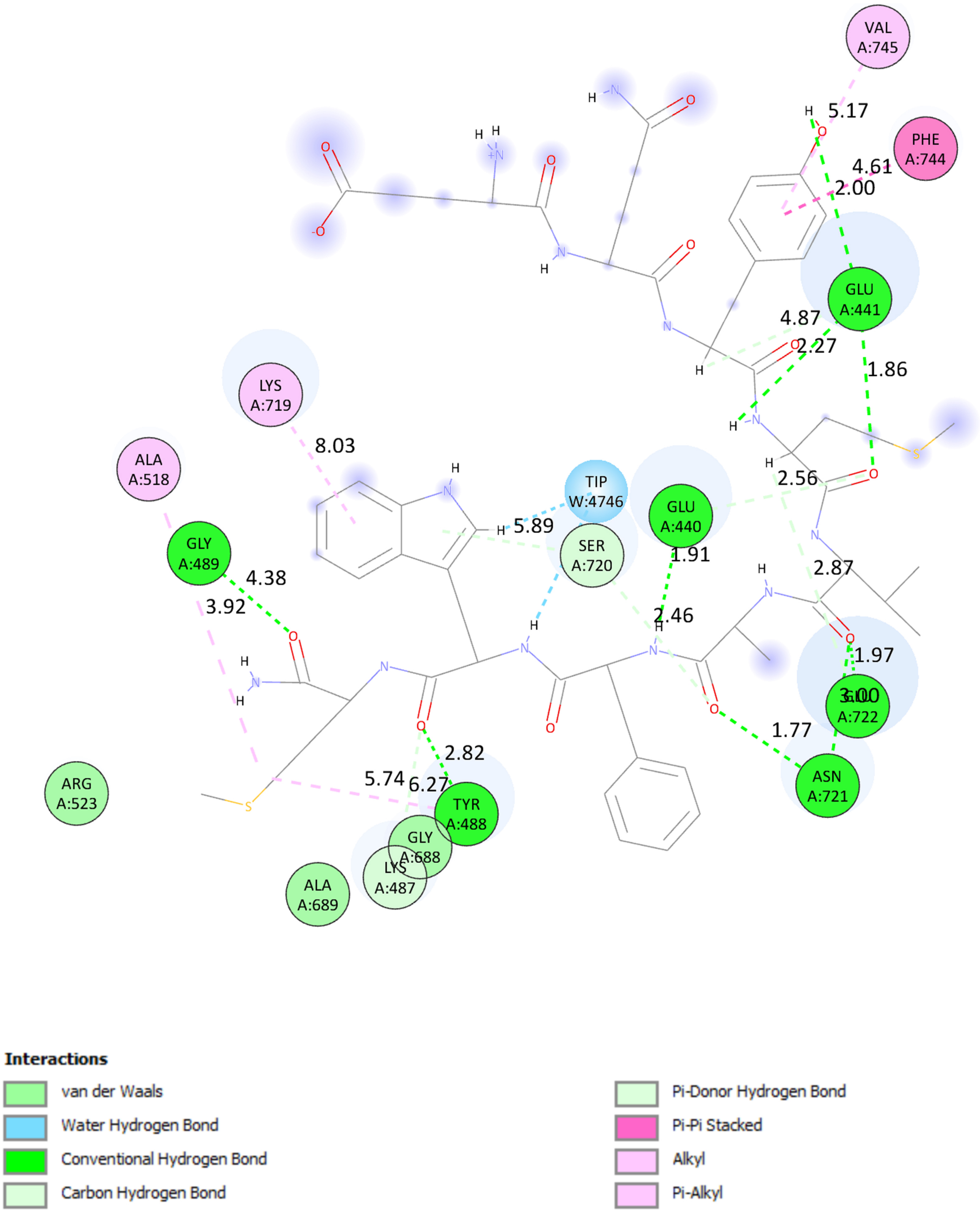

Supplementary Figure 9 legend: Interactions — van der Waals; Water Hydrogen Bond; Conventional Hydrogen Bond; Carbon Hydrogen Bond; Pi-Donor Hydrogen Bond; Pi-Pi Stacked; Alkyl; Pi-Alkyl

Supplementary Figure 9: Occidentalin-1202 interaction analysis after Molecular Dynamics. Interaction types vary according to the legend indications. Distances (Å) are shown close to the dashed lines.

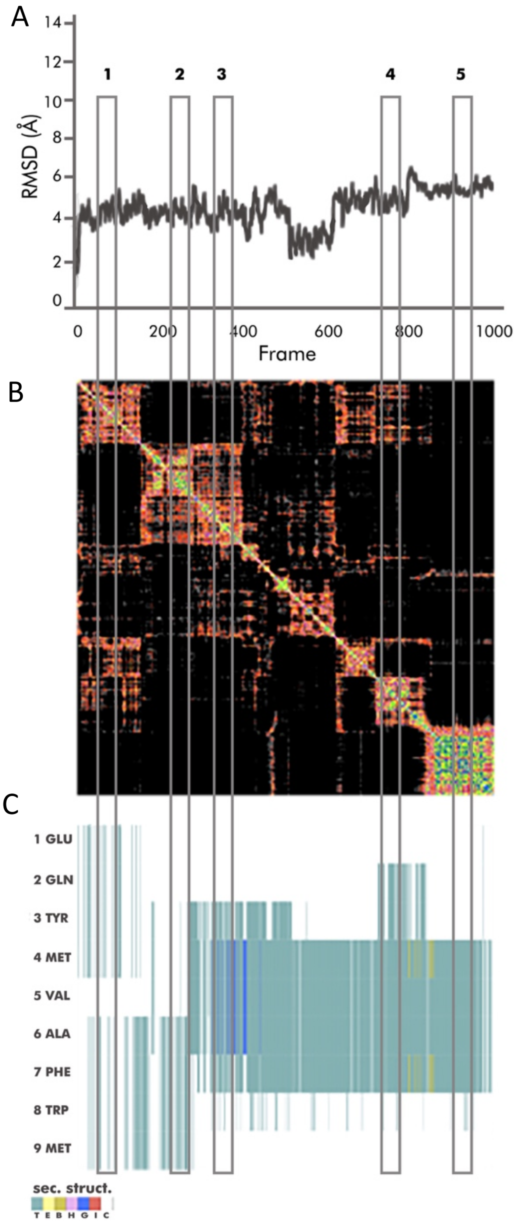

Supplementary Figure 10: Analysis by one-dimensional RMSD (A), two-dimensional RMSD (B) and analysis of secondary structures with the time of the peptide in a hydrophobic environment (C). Five conformations were more representative and original during the dynamics of 30 ns. In the legend, T: turn E: extended conformation; B: bridge; H: alpha-helix; G: helix 3-10; I: pi hélix; and C: coil.

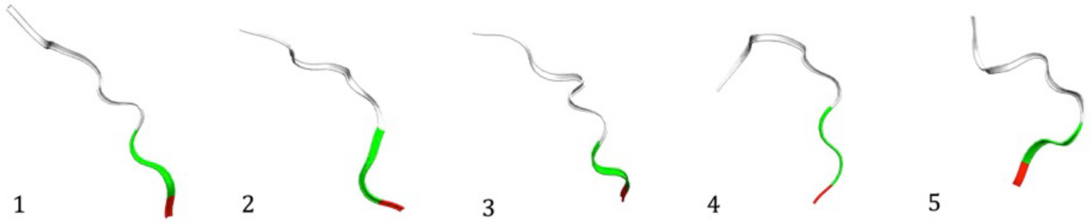

Supplementary Figure 11: Five representative and original conformations of the peptide Occidentalin in molecular dynamics of 30 ns in a hydrophobic environment.

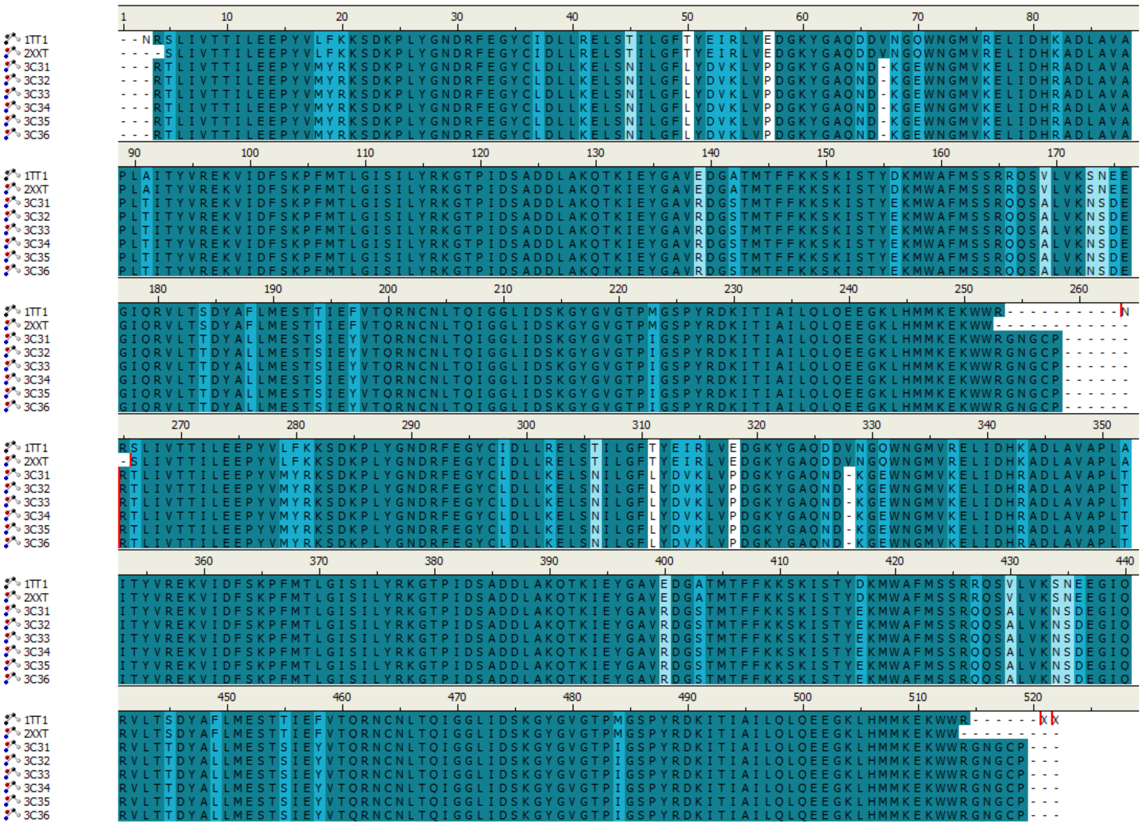

Supplementary Figure 12: Alignment of the eight kainate receptor structure sequences obtained from the PDB database. In dark blue, identical residues; in blue, the conserved substitutions; in light blue, the less conserved substitutions.

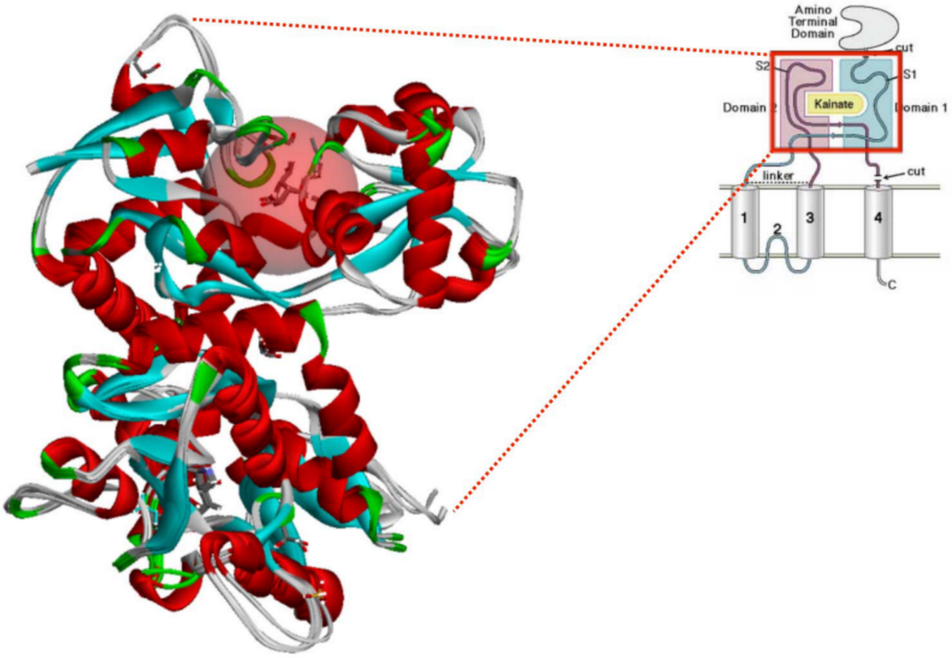

Supplementary Figure 13: Alignment of the eight kainate receptor structure sequences obtained from the PDB database.

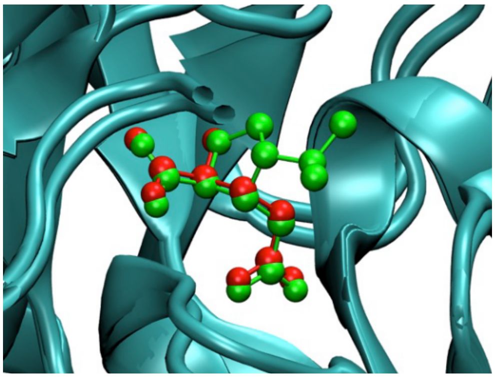

Supplementary Figure 14: Zoom of the glutamate and kainate agonist binding site connected to the GLUR6 receptor, after alignment of the structures referring to the PDB files 2XXR and 2XXT. PDB 2XXR, glutamate agonist (in red) linked to the binding site; PDB 2XXT, kainate agonist (in light green) linked to the binding site.

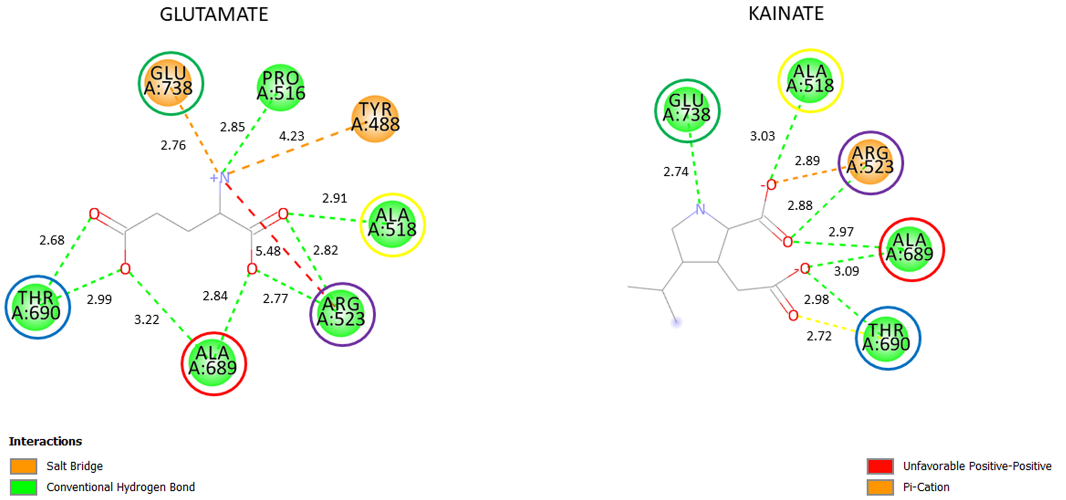

Supplementary Figure 15: Glutamate and Kainate interactions in GluR6 receptor (PDB: 2XXR and 2XXT respectively). Distances (Å) are shown close to the dashed lines.
